# Reversal of glucocorticoid resistance in paediatric acute lymphoblastic leukaemia is dependent on restoring *BIM* expression

**DOI:** 10.1038/s41416-020-0824-8

**Published:** 2020-04-03

**Authors:** Cara E. Toscan, Duohui Jing, Chelsea Mayoh, Richard B. Lock

**Affiliations:** Children’s Cancer Institute, School of Women’s and Children’s Health, UNSW Sydney, Sydney, NSW Australia

**Keywords:** Acute lymphocytic leukaemia, Paediatric cancer, Oncogenes, High-throughput screening, Gene expression

## Abstract

**Background:**

Acute lymphoblastic leukaemia (ALL) is the most common paediatric malignancy. Glucocorticoids form a critical component of chemotherapy regimens and resistance to glucocorticoid therapy is predictive of poor outcome. We have previously shown that glucocorticoid resistance is associated with upregulation of the oncogene *C-MYC* and failure to induce the proapoptotic gene *BIM*.

**Methods:**

A high-throughput screening (HTS) campaign was carried out to identify glucocorticoid sensitisers against an ALL xenograft derived from a glucocorticoid-resistant paediatric patient. Gene expression analysis was carried out using Illumina microarrays. Efficacy, messenger RNA and protein analysis were carried out by Resazurin assay, reverse transcription-PCR and immunoblotting, respectively.

**Results:**

A novel glucocorticoid sensitiser, 2-((4,5-dihydro-1*H*-imidazol-2-yl)thio)-*N*-isopropyl-*N*-phenylacetamide (GCS-3), was identified from the HTS campaign. The sensitising effect was specific to glucocorticoids and synergy was observed in a range of dexamethasone-resistant and dexamethasone-sensitive xenografts representative of B-ALL, T-ALL and Philadelphia chromosome-positive ALL. GCS-3 in combination with dexamethasone downregulated *C-MYC* and significantly upregulated *BIM* expression in a glucocorticoid-resistant ALL xenograft. The GCS-3/dexamethasone combination significantly increased binding of the glucocorticoid receptor to a novel *BIM* enhancer, which is associated with glucocorticoid sensitivity.

**Conclusions:**

This study describes the potential of the novel glucocorticoid sensitiser, GCS-3, as a biological tool to interrogate glucocorticoid action and resistance.

## Background

The introduction of multi-agent chemotherapy protocols in paediatric acute lymphoblastic leukaemia (ALL) has led to 5-year event-free survival rates of more than 85% and 5-year overall survival rates approaching 90% in developed countries.^1–4^ However, due to the high incidence of paediatric ALL, it remains one of the most common causes of death from disease in children, and relapsed ALL has a similar incidence to rhabdomyosarcoma.^5–7^ Glucocorticoids (e.g. dexamethasone, prednisolone) are critical components of chemotherapy protocols in paediatric ALL, and response to glucocorticoid therapy is a major prognostic factor.^8–10^ Contemporary clinical trials based on the Berlin–Frankfurt–Munich (BFM) protocol include an initial week of glucocorticoid monotherapy, prior to multi-agent chemotherapy, where poor response is predictive of an inferior outcome.^10–12^ Acquired resistance to glucocorticoids is common at relapse, and is disparate compared with other chemotherapeutic agents used in the treatment of paediatric ALL.^13,14^ Although glucocorticoids can be extremely effective, the emergence of intrinsic and acquired resistance remains a significant barrier to cure paediatric ALL.

Glucocorticoids are steroid hormones that induce apoptosis in normal and malignant lymphoid cells.^15,16^ Glucocorticoids enter the cell through passive diffusion, where they bind to the glucocorticoid receptor (GR), a member of the nuclear receptor family of ligand-dependent transcription factors.^15,16^ Once activated, the GR translocates to the nucleus where it activates target genes by binding as a homodimer to specific palindromic DNA sequences, known as glucocorticoid response elements (GREs).^15,16^ We have previously demonstrated that dexamethasone resistance in ALL xenografts and patient samples occurred downstream of GRE binding, and that dexamethasone resistance was associated with failure to induce the proapoptotic protein BIM.^17,18^ We have found that the *BIM* gene is epigenetically silenced via deacetylation of histone H3 in dexamethasone-resistant ALL xenografts, and in two independent cohorts of chemo-resistant patient biopsy samples.^19^

Historically, little is known about the signalling pathways that regulate *BIM* expression after glucocorticoid treatment. Recent work in our laboratory has identified two novel signalling pathways involved in glucocorticoid-induced apoptosis of ALL cells.^20,21^ In the first signalling pathway, the activated GR induces *KLF13* expression by binding to its promoter. KLF13 inhibits SP1-triggered *MYB* transcription, and *MYB* suppression leads to decreased expression of the anti-apoptotic protein, BCL-2. However, in dexamethasone-resistant ALL, treatment with glucocorticoids activates the GR, but *KLF13* expression is not induced and apoptosis is inhibited. In the second signalling pathway, the activated GR binds directly to an intronic region in the *BIM* gene (*BIM*-IGR), triggering *BIM* expression, which results in apoptosis. In dexamethasone-resistant ALL, the activated GR cannot bind to the *BIM*-IGR to trigger apoptosis. In a separate study, we have shown that in vivo dexamethasone treatment in a glucocorticoid-sensitive ALL xenograft caused significant repression of the oncogene *C-MYC*.^22^

We have performed a 40,000 compound high-throughput screening (HTS) campaign to identify glucocorticoid sensitisers specifically designed to reverse dexamethasone resistance in the paediatric ALL xenograft, ALL-19.^23^ ALL-19 was selected for use in the screen as it exhibits robust and high-level dexamethasone resistance both ex vivo and in vivo, and is representative of the most common paediatric ALL subtype, B-ALL.^24,25^ The screen identified four lead dexamethasone sensitisers, which all contained a thioimidazoline moiety.^26^ We developed structure–activity relationships for 32 thioimidazoline-based compounds and identified 2-((4,5-dihydro-1*H*-imidazol-2-yl)thio)-*N*-isopropyl-*N*-phenylacetamide (GCS-3) as the lead glucocorticoid sensitiser due to its superior ex vivo half-life in liver microsomes.^26^ In this study, we show that GCS-3 downregulates *C-MYC* and reverses *BIM* repression through both signalling pathways in a glucocorticoid-resistant ALL xenograft. We demonstrate the applicability of GCS-3 as a biological tool to further elucidate the mechanisms of glucocorticoid resistance and the development of strategies to reverse resistance in paediatric ALL.

## Methods

Information on reagents is provided in the Supplementary Methods section.

### Ex vivo culture of xenograft and non-leukaemic cells

The development and characterisation of a series of paediatric ALL xenografts derived from patient biopsies have been previously described.^24,25^ All assays were performed using authenticated stocks of xenograft cells.^27^ Human peripheral blood mononuclear cells (PBMCs) and CD34^+^ cells isolated from human cord blood were purchased (Lonza, VIC, Australia) or obtained from the Sydney Cord Blood Bank under approval of the Prince of Wales Hospital. Mononuclear cells were separated from cord blood by density gradient centrifugation (ELITech Australian and New Zealand, VIC, Australia) and CD34^+^ cells were isolated using magnetic beads and autoMACS separation (Miltenyi Biotec, NSW, Australia). Culture of CD34^+^ cells was carried out as previously described.^28^

For experiments using xenograft cells and PBMCs, cells were retrieved from cryostorage and resuspended in RPMI-1640 medium (Invitrogen Life Technologies, Gaithersburg, MD) supplemented with 10% foetal bovine serum (Invitrogen Life Technologies) (complete RPMI). Cells were centrifuged, aspirated, and washed with complete RPMI. The cells were resuspended in QBSF-60 medium (Quality Biological, Gaithersburg, MD) supplemented with Flt-3 ligand (Amgen, Thousand Oaks, CA) (complete QBSF) at a cell concentration previously optimised for each xenograft/peripheral blood mononuclear cell (1–5 × 10^6^ cells per mL). Viability was determined by the exclusion of 0.2% trypan blue (Sigma, NSW, Australia). For all experiments, cells were seeded and equilibrated overnight at 37 °C, 5% CO_2_ prior to drug treatment.

### Cytotoxicity assays

In both single agent and combination cytotoxicity assays, 100 µL of cell suspension was seeded in 96-well clear, U-bottom tissue culture-treated plates (In Vitro Technologies, VIC, Australia) at a cell concentration previously optimised. Plates were equilibrated overnight at 37 °C, 5% CO_2_ prior to drug treatment. Compounds were serially diluted in complete QBSF medium and added in triplicate wells. In combination cytotoxicity assays, cells were treated with GCS-3 and dexamethasone simultaneously at a fixed ratio of concentrations corresponding to 0.25, 0.5, 1, 2, and 4 times the half-maximal inhibitory concentration (IC_50_) values for each drug independently and in combination. Where the IC_50_ values were >10 µM, cells were treated with the following concentrations: 2.5, 5, 10, 20 and 40 µM, independently and in combination. Following 48 h incubation (unless otherwise stated) at 37 °C, 5% CO_2_, cell viability was assessed by mitochondrial activity assay (Resazurin cell viability assay, 6 h incubation) or by flow cytometry (7-aminoactinomycin D and Annexin V; BD Biosciences, NSW, Australia). Cell viability was calculated as the percentage of untreated controls. IC_50_ values were calculated from cumulative survival curves.

### Calculation of combination effect

To determine whether GCS-3 was synergistic, additive or antagonistic with dexamethasone, the Bliss-Additivity (BA) model was used.^29^ Deviation from BA was calculated at each tested dose, where synergy is defined as ≥0.05 and antagonism as ≤−0.05 (Table 1).

**Table 1:** Combination effect descriptions.

Deviation from BA	Description
≥0.05	Synergy
0.04 to −0.04	Nearly additive
≤−0.05	Antagonism

### Illumina gene expression and analysis

Illumina gene expression arrays were performed by the Ramaciotti Centre (UNSW Sydney, NSW, Australia). Microarray studies were performed on complementary RNA samples using the Illumina Direct Hybridisation Gene Expression Array (Illumina, San Diego, CA). The studies were performed using HumanHT-12 v4 BeadChips (12 samples per chip) from Illumina. Samples were performed in duplicate and data were processed in R using Limma (a Bioconductor package). Data were normalised using neqc and bad quality probes were removed from further analysis. Differential expression analysis was performed to identify over-/under-expressed genes at 12 and 24 h (fold-change ≥ |2|, *p* < 0.05) when comparing treatment with dexamethasone, GCS-3 and GCS-3/dexamethasone combination therapy against control. Significant genes were further analysed in KEGG (Kyoto Encyclopaedia of Genes and Genomes) pathway analysis and Gene Ontology (GO; Biological Processes, Cellular Components and Molecular Function) enrichment using DAVID. Functional analysis of the differentially expressed genes between dexamethasone and combination treatments was performed using gene set enrichment analysis [preRanked gene set enrichment analysis (GSEA), Broad Institute].

### Real-time quantitative reverse transcription-PCR

Real-time quantitative reverse transcription-PCR (qRT-PCR) was performed using standard techniques. RNA was isolated using TRIzol (Invitrogen Life Technologies) and purified using the RNeasy RNA Isolation Kit (Qiagen, Germantown, MD). First-strand complementary DNA was synthesised using MMLV reverse transcriptase (Invitrogen Life Technologies). TaqMan primers and probes for *KLF13* (Hs00429818_m1), *BCL-2* (Hs00608023_m1), *BIM* (*BCL2L11*, Hs00708019_s1), *GR* (*NR3C1*, Hs00230813_m1), *MYB* (Hs00920556_m1) *MYC* (Hs00153408_m1) and *EF1α* (elongation factor 1α; Hs00265885_g1) were purchased from Invitrogen Life Technologies and RT-PCR was carried out in duplicate under cycling conditions according to the manufacturer’s instructions. *EF1α* was used as an internal control for each sample, where the primers and probe were optimised to not interfere with target gene amplification (primers *EF1α* forward, 5′-CTGAACCATCCAGGCCAAAT-3′; *EF1α* reverse, 5′-GCCGTGTGGCAATCCAAT-3′; probe, 5′-VIC-AGCGCCGGCTATGCCCCTG-TAMRA-3′). Target messenger RNA expression was normalised to *EF1α* in each sample and fold differences were calculated by comparison with vehicle-treated controls using the ΔΔCt method.

### Immunoblotting

Whole-cell lysates of ALL-19 xenograft cells were prepared by incubation with RIPA lysis buffer supplemented with Protease Inhibitor (Sigma). Nuclear and cytoplasmic cell lysates were prepared using the NE-PER Nuclear and Cytoplasmic Extraction Kit (Invitrogen Life Technologies). Protein concentration was determined by BCA assay using a bovine serum albumin standard (Invitrogen Life Technologies). The membranes were probed with primary antibodies: BIM, MYC, GR (Cell Signalling, Danvers, MA), actin, α-tubulin (Sigma), DNA topoisomerase I (Novus Biologicals, Centennial, CO). Secondary antibodies were horseradish peroxidase conjugates of either anti-mouse or anti-rabbit immunogIobulin G (IgG) (Sigma). The bound secondary antibody was detected by chemiluminescence and imaged using a VersaDoc Imaging System (Model 5000).

### GR-DNA-binding ELISA

Nuclear cell lysates were assayed for GR-DNA-binding activity using the TransAM Transcription Factor ELISA (enzyme-linked immunosorbent assay) Kit (Active Motif, Carlsbad, CA). GR-DNA-binding activity was expressed relative to a HeLa-positive control nuclear lysate, prepared from HeLa cells treated with 100 nM dexamethasone for 1 h (supplied in the kit).

### Chromatin immunoprecipitation

Chromatin immunoprecipitation (ChIP) was carried out as previously described.^20^ Briefly, xenograft cells were treated with vehicle control, 1 µM dexamethasone, 10 µM GCS-3 or a combination of 1 µM dexamethasone and 10 µM GCS-3 for 8 h ex vivo, and fixed with 1% formaldehyde for 10 min at room temperature. Nuclei were extracted from fixed cells by 10 min incubation in lysis buffer (0.2% NP40 in 10 mM Tris buffer, pH 8.0), followed by centrifugation at 1250 × *g* for 5 min at 4 °C. Chromatin was fragmented using a Bioruptor sonicator (Diagenode SA, Belgium) on high power at 4 °C with 30 s on/off for 10 min. Separate immunoprecipitates were produced using Ig raised against the GR (Cell Signalling) and processed according to the manufacturer’s instructions. DNA from protein-associated complexes and corresponding input samples was recovered using phenol/chloroform/isoamyl alcohol with phase lock gel tubes (5 Prime, Hilden, Germany). The ChIP DNA samples were assayed by SYBR-green real-time PCR using custom primers (Table 2) and the ABI7900 PCR instrument (Life Technologies, CA). The fold enrichment of ChIP DNA with target antibodies was normalised to IgG control (IgG from rabbit serum, Sigma).

**Table 2:** *BIM*-IGR, *GILZ* R1 and *GILZ* R2 primer sequences.

	Forward primer	Reverse primer
*BIM*-IGR	CCAACTACTGGTGCCTCACA	CCCACCTGCCACTTCTGAAA
*GILZ* R1	AGCCATGAACACCGAAATG	TCCAGCTTAACGGAAACCAC
*GILZ* R2	ATGTGGTTTAACTGGGCCAC	ATATAGGAGGAGCCGGCTG

### Luciferase reporter assay

The luciferase reporter assay was performed using pGL2B vectors from Promega (Madison, WI). *BIM* promoter and IGR sequences were synthesised as double-stranded DNA (dsDNA) by Integrated DNA Technologies (Coralville, IA). The genomic location and size of dsDNA are listed in Table 3. The promoter dsDNA was inserted into pGL2B vector at the multiple cloning region 20 bp upstream of the 5′ end of firefly luciferase gene, and the IGR dsDNA was inserted at 1 kb downstream of the 3′ end of the firefly luciferase gene. The cloned vector was linearised by *Sca*I restriction enzyme, and a PGKNeo (Promega) vector was linearised by *Hin*dIII. The two linearised vectors were co-transfected into Nalm6 cells and incubated at 37 °C overnight. G418 (Geneticin, Sigma) was added to the transfected cells at 1 mg/mL and incubated for 7–10 days. The firefly luminescence was detected in the G418-selected cells after 16 h treatment with vehicle control, 1 µM dexamethasone, 10 µM GCS-3 or a combination of 1 µM dexamethasone and 10 µM GCS-3 using the Bright-Glo™ Luciferase Assay System (Promega). Fold inductions were calculated by normalising to vehicle control.

**Table 3:** Genomic location of *BIM* promoter and *BIM*-IGR.

Cloned sequences	Genomic location	Size (bp)
*BIM* promoter	Chr2: 111,877,900–111,878,679	780
*BIM*-IGR	Chr2: 111,914,645–111,915,877	1233

## Results

### Ex vivo sensitivity of ALL xenografts to dexamethasone correlates with patient outcome

The development and characterisation of paediatric ALL xenografts derived from patient biopsies have been previously described in detail.^24,25^ We assessed the ex vivo dexamethasone sensitivity of a panel of 20 ALL xenografts, which consisted of a range of ALL subtypes, including: B-ALL, T-ALL, Philadelphia chromosome-positive ALL (Ph^+^-ALL), mixed lineage leukaemia-rearranged (*MLL*r) and early T-cell precursor ALL (ETP-ALL). The ALL xenografts were established from patient samples taken at either diagnosis or relapse, and the current clinical status varies from alive in 1–3rd complete remission (CR1–3) to died of disease (DOD). Two distinct groups of dexamethasone-sensitive (IC_50_ < 100 nM) and dexamethasone-resistant (IC_50_ > 40 µM) were observed (Supplementary Table S1). A significant correlation was observed between ex vivo dexamethasone efficacy (IC_50_) and patient clinical status (CR1–3 or DOD) in the 19 ALL xenografts where clinical data were available (Supplementary Fig. S1). No significant correlations were observed between ex vivo dexamethasone sensitivity and ALL subtype or disease status at biopsy (data not shown). This finding highlights the clinical relevance of the ALL xenograft model in studying glucocorticoid resistance.

### GCS-3 is a specific glucocorticoid sensitiser

**Fig. 1:**
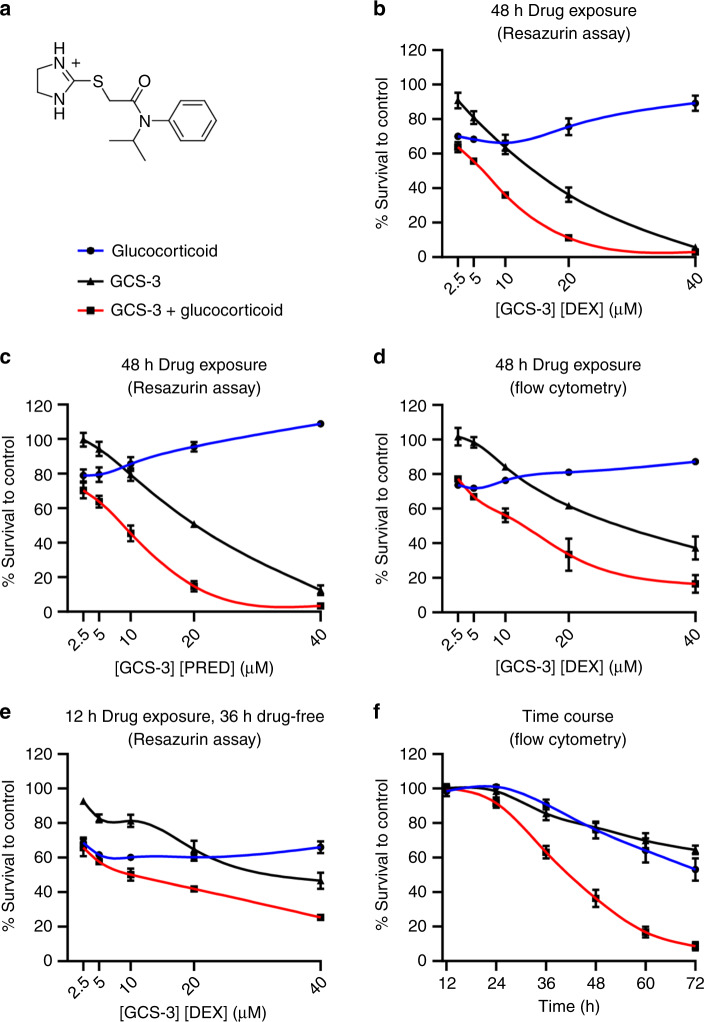
Ex vivo efficacy of GCS-3 in combination with dexamethasone or prednisolone against ALL-19 xenograft cells. **a** Chemical structure of GCS-3, 2-((4,5-dihydro-1*H*-imidazol-2-yl)thio)-*N*-isopropyl-*N*-phenylacetamide. **b**–**d** ALL-19 xenograft cells were exposed to GCS-3, glucocorticoid or both in combination at a fixed ratio of concentrations for 48 h, and cell viability was then assessed by Resazurin assay (**b**, **c**) or flow cytometry (**d**). **e** After 12 h drug incubation, drug-containing media were removed and cells were incubated in fresh media for 36 h, and cell viability was assessed by Resazurin assay. **f** ALL-19 xenograft cells were treated with 10 µM GCS-3, 10 µM dexamethasone or both in combination. Cell viability was determined by flow cytometry at various time points up to 72 h. Each data point represents the mean ± SEM of at least three independent experiments.

The chemical structure of the novel glucocorticoid sensitiser GCS-3 is shown in Fig. 1a, and its synthesis and characterisation have been reported previously.^26^ GCS-3 was identified from an HTS campaign to reverse dexamethasone resistance in the paediatric ALL xenograft, ALL-19, where compounds were added simultaneously.^23^ Modifying the timing of GCS-3 addition confirmed that dexamethasone sensitisation was maximal when GCS-3 and dexamethasone were administered simultaneously (Supplementary Fig. S2). All experiments henceforth have compounds added simultaneously unless otherwise stated.

Fixed-ratio combination cytotoxicity assays showed that GCS-3 was synergistic with dexamethasone or prednisolone against ALL-19; hence, GCS-3 is a broad glucocorticoid sensitiser (Fig. 1b, c, Supplementary Table S2). Similar results were observed when the GCS-3/dexamethasone combination was tested against ALL-19 using a direct measure of cell viability (Fig. 1d, Supplementary Table S2). The action of GCS-3 is reversible as removal of GCS-3/dexamethasone after 12 h incubation against ALL-19 resulted in loss of single agent and combination activity (Fig. 1e, Supplementary Table S2). A time-course experiment against ALL-19 showed that the combination caused a marked decrease in cell viability compared to the single agents, with <10% viable cells remaining at 72 h (Fig. 1f).

To determine the effect of GCS-3/dexamethasone on non-leukaemic cells, fixed-ratio combination cytotoxicity assays were performed on human PBMCs and human CD34^+^ cells. The GCS-3/dexamethasone combination was not synergistic in normal human cells (Supplementary Figs. S3 and S4, Supplementary Table S3). To determine if GCS-3 is a specific glucocorticoid sensitiser, fixed-ratio combination cytotoxicity assays were performed with GCS-3 in combination with other classes of chemotherapeutics (daunorubicin, vincristine or cisplatin). GCS-3 was not synergistic in combination with all three chemotherapeutic drugs at every concentration tested in ALL-19 cells (Supplementary Fig. S5, Supplementary Table S4). This suggests that GCS-3 selectively restores glucocorticoid sensitivity.

### GCS-3 has broad applicability

**Fig. 2:**
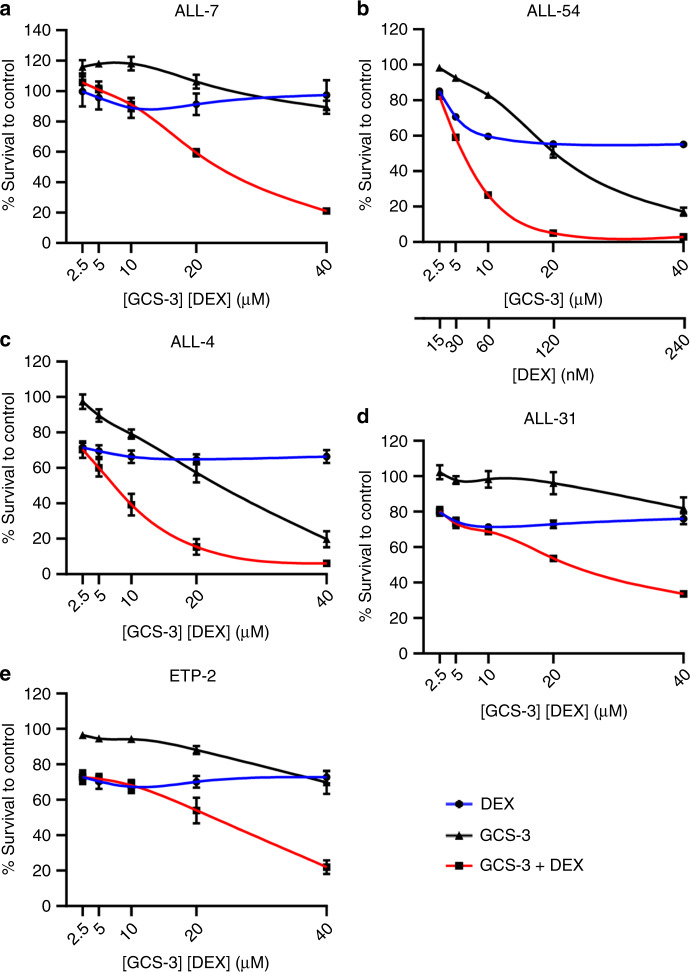
Ex vivo efficacy of GCS-3 in combination with dexamethasone against ALL xenograft cells. ALL-7 (**a**), ALL-54 (**b**), ALL-4 (**c**), ALL-31 (**d**) and ETP-2 (**e**) xenograft cells were exposed to GCS-3, dexamethasone or both in combination at a fixed ratio of concentrations for 48 h, and cell sensitivity was then assessed by Resazurin cytotoxicity assay. Each data point represents the mean ± SEM of at least three independent experiments.

To determine if GCS-3 showed broader applicability as a glucocorticoid sensitiser, fixed-ratio combination cytotoxicity assays were performed with dexamethasone against an additional 19 ALL xenografts (Fig. 2, Supplementary Fig. S6, Supplementary Table S5). Thirteen B-ALL xenografts including five dexamethasone-sensitive xenografts (ALL-54, ALL-17, ALL-55, ALL-56 and ALL-3) and eight dexamethasone-resistant xenografts (ALL-19, ALL-2, ALL-50, ALL-7, ALL-88, ALL-84, ALL-4 and MLL-5) were tested. The greatest sensitising effect was observed in ALL-7 (Fig. 2a), which is a high-risk subtype with a *TCF3-HLF* fusion gene, where the highest concentration of GCS-3/dexamethasone caused 66% greater cytotoxicity than the calculated additive effect. In the high-risk Ph^+^ B-ALL subtype, sensitising effects were observed in the highly dexamethasone-resistant xenograft, ALL-4 (Fig. 2c). In summary, GCS-3 sensitised 5 out of 13 B-ALL xenografts to dexamethasone, including dexamethasone-resistant and dexamethasone-sensitive xenografts.

Seven T-ALL xenografts including three dexamethasone-sensitive xenografts (ALL-43, ALL-16, ETP-1) and four dexamethasone-resistant xenografts (ALL-31, ALL-32, ETP-2, ETP-3) were tested. The greatest sensitising effect was observed in ALL-31 (Fig. 2d), where the highest concentration of GCS-3/dexamethasone caused 29% greater cytotoxicity than the calculated additive effect. In the high-risk ETP-ALL subtype, sensitising effects were observed in the dexamethasone-resistant xenograft, ETP-2 (Fig. 2e). In summary, GCS-3 sensitised two out of seven T-ALL xenografts to dexamethasone. GCS-3 showed broad applicability on multiple ALL subtypes to restore or enhance glucocorticoid sensitivity. Overall, synergy was observed in 6 out of 12 dexamethasone-resistant and 1 out of 8 dexamethasone-sensitive xenografts.

### GCS-3 requires a functional GR to induce caspase-dependent apoptosis

**Fig. 3:**
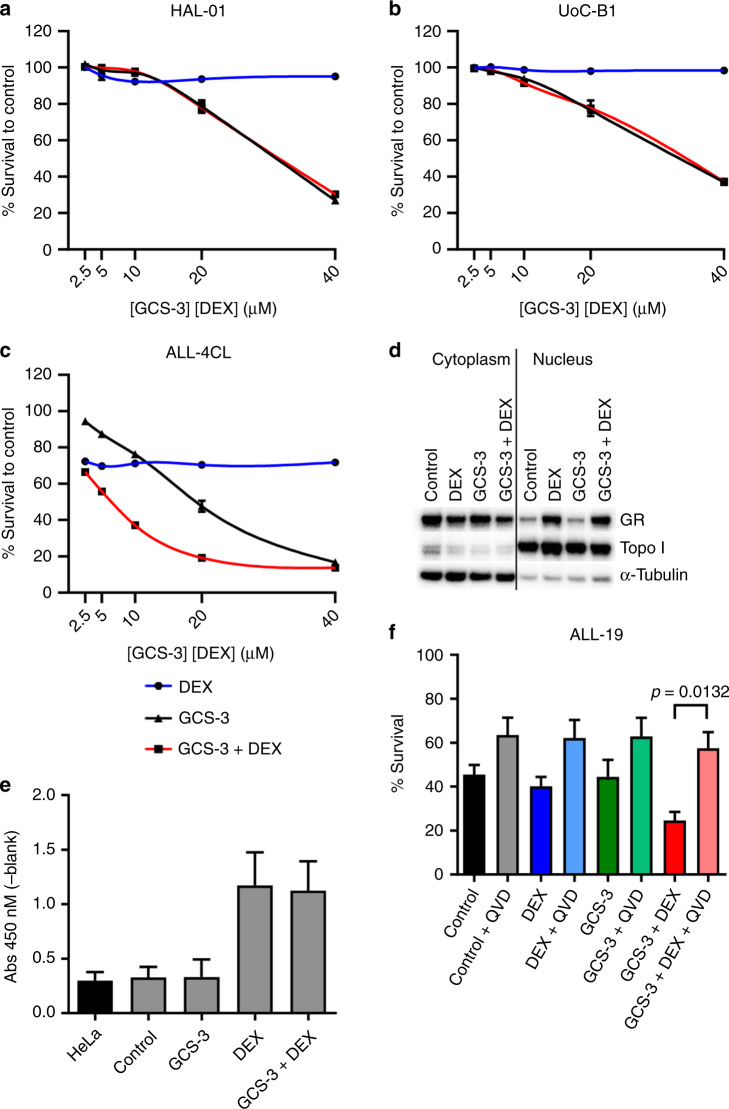
GCS-3 requires a functional GR to induce caspase-dependent apoptosis. HAL-01 (**a**), UoC-B1 (**b**) or ALL-4CL (**c**) cells were exposed to GCS-3, dexamethasone or both in combination at a fixed ratio of concentrations for 48 h, and cell sensitivity was then assessed by Resazurin cytotoxicity assay. Each data point represents the mean ± SEM of at least three independent experiments. **d**, **e** ALL-19 xenograft cells were treated with 10 µM GCS-3 and 1 µM dexamethasone (DEX) for 1 h before separation into nuclear and cytoplasmic fractions. **d** Equal amounts of protein (10 µg) from each fraction were immunoblotted for GR, Topo I (nuclear loading control) and α-tubulin (cytoplasm loading control). **e** Dexamethasone-induced binding of the GR to a GRE motif was assessed using a DNA-binding ELISA, with equal amounts of nuclear lysates and HeLa control lysate (5 µg). The OD450 nm of each sample minus blank is shown. Each data point represents the mean ± SEM of three independent experiments. **f** ALL-19 xenograft cells were pre-treated with 10 µM QVD-OPh (QVD) or vehicle control for 2 h. Cells were then exposed to 10 µM GCS-3, 10 µM dexamethasone (DEX) or both for 48 h and cell sensitivity was assessed by flow cytometry. Each data point represents the mean ± SEM of four independent experiments. Significance was calculated using the unpaired *t* test with Welch’s correction.

To determine if GCS-3 required a functional GR to sensitise ALL cells to glucocorticoids, GCS-3 in combination with dexamethasone was assessed in two glucocorticoid-resistant BCP-ALL cell lines, HAL-01 and UoC-B1. Both cell lines are highly dexamethasone-resistant, but unlike glucocorticoid-resistant xenografts and patient biopsies, both cell lines express a dysfunctional GR.^18^ The GCS-3/dexamethasone combination was nearly additive in both cell lines (Fig. 3a, b, Supplementary Table S6). To determine if GCS-3 was able to sensitise proliferating ALL cells to glucocorticoids, the GCS-3/dexamethasone combination was tested in the glucocorticoid-resistant B-ALL cell line, ALL-4CL. The ALL-4CL is derived from the ALL-4 xenograft and maintains resistance to glucocorticoids while expressing a functional GR.^18^ As seen with the ALL-4 xenograft, the GCS-3/dexamethasone combination was synergistic in ALL-4CL (Fig. 3c, Supplementary Table S6). Although GCS-3 requires a functional GR to sensitise ALL cells to glucocorticoids, the addition of GCS-3 did not enhance dexamethasone-induced GR translocation to the nucleus or GR-DNA binding in ALL-19 cells (Fig. 3d, e, respectively, Supplementary Fig. S7).

Both glucocorticoids and vincristine induce cell death in lymphoid cells via caspase-dependent apoptosis.^30^ To determine whether this cell death mechanism was activated by the GCS-3/dexamethasone combination in ALL-19 cells, the effect of the pan-caspase inhibitor QVD-OPh on the combination treatment was assessed.^31^ As the glucocorticoid-resistant xenograft ALL-19 is vincristine sensitive, vincristine was used as a positive control (Supplementary Fig. S8). Pre-incubation with QVD-OPh significantly increased the viability of ALL-19 cells treated with the combination (Fig. 3f). Therefore, the combination of GCS-3 and dexamethasone induces caspase-dependent apoptosis in ALL-19 xenograft cells and this effect can be inhibited using the pan-caspase inhibitor, QVD-OPh.

### GCS-3 downregulates *C-MYC* expression

To gain insight into the mechanism of action of GCS-3 in combination with dexamethasone, microarray analysis of gene expression was performed on ALL-19 cells treated with vehicle, GCS-3, dexamethasone or the combination for 12 or 24 h (Supplementary Fig. S9). Unsupervised hierarchical clustering demonstrated that there are few differences in the expression profile between GCS-3 and the vehicle at either time point, which was confirmed with differential expression analysis (Supplementary Table S7, Supplementary Fig. S10). Treatment with the combination of GCS-3 and dexamethasone caused the largest change in the gene expression profile of ALL-19 cells, with 1049 genes differentially expressed after 24 h treatment (*p* < 0.05, FC ≥ |2|; Supplementary Table S7).

**Fig. 4:**
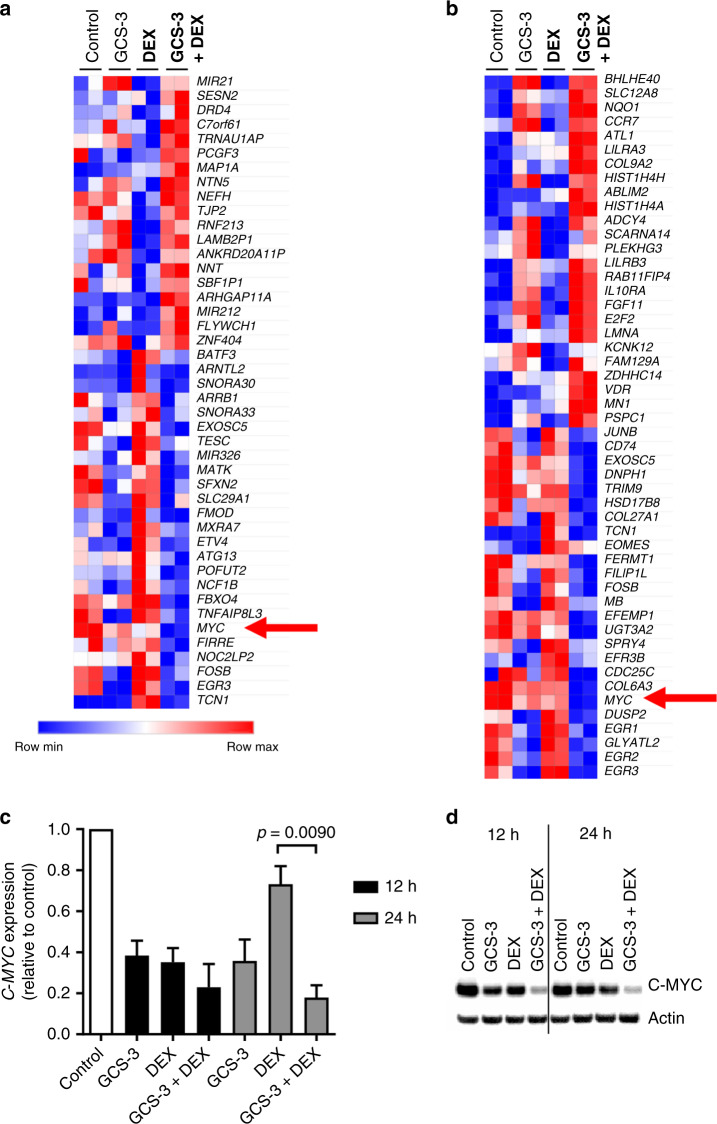
Effects of GCS-3 on dexamethasone-induced gene expression changes. ALL-19 cells were treated with 10 µM dexamethasone (DEX), 10 µM GCS-3 or both in combination and gene expression profiling was performed. The top differentially expressed genes between the dexamethasone and combination treatment groups are shown after 12 h (**a**) and 24 h (**b**). Expression levels were normalised for each gene, where the mean is 0, higher than the mean are shown in red and lower in blue. **c** *C-MYC* mRNA expression was analysed by qRT-PCR and calculated relative to the 12 and 24 h vehicle-treated controls. Each data point represents the mean ± SEM of three independent experiment. Significance was calculated using the unpaired *t* test with Welch’s correction. **d** C-MYC protein expression was analysed after 12 and 24 h treatment by immunoblotting and a representative blot is shown.

To identify genes induced or repressed by GCS-3 in the presence of dexamethasone, differential gene expression was performed between the dexamethasone and combination treated groups. The 50 most differentially expressed genes after 12 and 24 h treatment are presented in Fig. 4a, b. *C-MYC* was one of the top 25 downregulated genes in the combination group compared to either single agent (Fig. 4a, b). This was confirmed by PCR and immunoblotting, where downregulation of *C-MYC* by the GCS-3/dexamethasone combination was 2.0-fold greater than GCS-3 alone and 4.1-fold greater than dexamethasone alone at 24 h (Fig. 4c, d, Supplementary Fig. S11).

GO analysis of the genes downregulated by GCS-3 in the presence of dexamethasone clustered into key biological processes and molecular functions that involved *C-MYC*, such as gene transcription (Supplementary Fig. S12A, B). GSEA was performed on the differentially expressed genes between dexamethasone and the combination treated groups at 24 h to determine whether genes induced and repressed by GCS-3 in the presence of dexamethasone are significantly concordant with published gene sets. The gene sets generated by Rhein et al.^32^ depict the changes in gene expression that occurred in ALL patients upon treatment with glucocorticoids in vivo. Genome-wide expression analysis was performed on blasts from 18 B-ALL patients at days 0 and 8, after prednisone treatment. GSEA revealed that genes upregulated/downregulated by GCS-3 in the presence of dexamethasone in ALL-19 cells were highly enriched for genes upregulated/downregulated after glucocorticoid treatment in B-ALL patients (Supplementary Fig. S12C, D). These results show that the gene expression changes caused by GCS-3 in the presence of dexamethasone in ALL-19 cells are relevant to what is observed by glucocorticoid treatment in a clinical setting.

### GCS-3 restores *BIM* expression

**Fig. 5:**
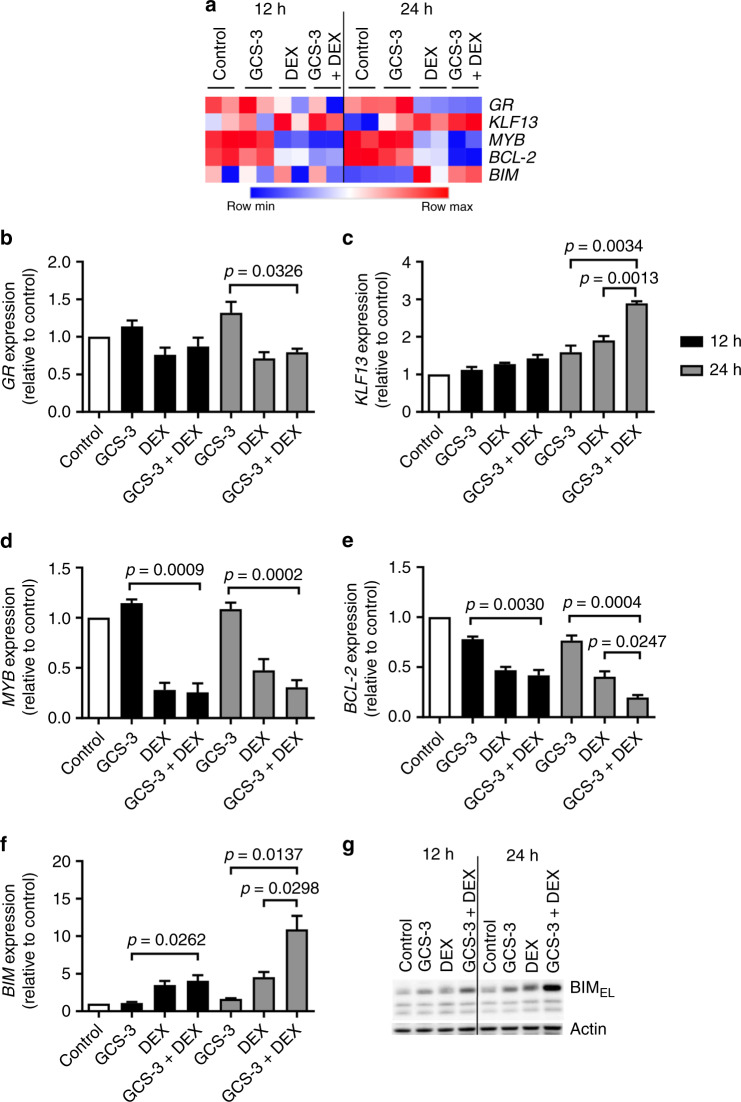
Expression levels of candidate genes in ALL-19 cells. **a** Microarray analysis of the effects of 10 µM GCS-3, 10 µM dexamethasone and the combination on gene expression in ALL-19 cells. Two biological replicates were analysed at 12 and 24 h post treatment, where red represents high and blue low gene expression. *GR* (**b**), *KLF13* (**c**), *MYB* (**d**), *BCL-2* (**e**) and *BIM* (**f**) mRNA expression was analysed by qRT-PCR and calculated relative to the 12 and 24 h vehicle-treated controls. Each data point represents the mean ± SEM of four independent experiments. Significance was calculated using the unpaired *t* test with Welch’s correction. **g** BIM protein expression was analysed after 12 and 24 h treatment by immunoblotting and a representative blot is shown.

Recent work in our laboratory has identified two novel signalling pathways involved in glucocorticoid-induced apoptosis in ALL cells, and both centre on the opposing regulation of the proapoptotic gene, *BIM*, and the anti-apoptotic gene, *BCL-2*.^20^ GCS-3 was utilised to determine whether these two novel signalling pathways can be reactivated in ALL-19 cells. The glucocorticoid-induced apoptotic response in lymphoid cells is mediated through the GR. Microarray and PCR analysis of *GR* expression after 12 and 24 h treatment showed that the GCS-3/dexamethasone combination did not reverse the dexamethasone-induced downregulation of *GR* expression (Fig. 5a, b). In glucocorticoid-sensitive ALL cells, treatment with glucocorticoids activates the *GR*, which up-regulates *KLF13* expression, which is abrogated in glucocorticoid-resistant ALL.^20^ The GCS-3/dexamethasone combination significantly increased *KLF13* expression compared to either single agent at 24 h (Fig. 5a, c). In glucocorticoid-sensitive ALL cells *KLF13* displaces the *MYB* activator, *SP1*, and represses *MYB* expression. The GCS-3/dexamethasone combination further downregulated *MYB* expression compared to either single agent at 24 h (Fig. 5a, d). In glucocorticoid-sensitive ALL cells, *MYB* suppression leads to decreased expression of anti-apoptotic *BCL-2*. The GCS-3/dexamethasone combination significantly decreased *BCL-2* expression by 3.9-fold compared to GCS-3 alone and 2.1-fold compared to dexamethasone alone at 24 h (Fig. 5a, e). In glucocorticoid-sensitive ALL cells, *BCL-2* suppression allows *BAX*-mediated apoptosis. The GCS-3/dexamethasone combination significantly increased *BIM* expression by 6.6-fold compared to GCS-3 alone and 2.4-fold compared to dexamethasone alone at 24 h (Fig. 5a, f, g, Supplementary Fig. S13).

**Fig. 6:**
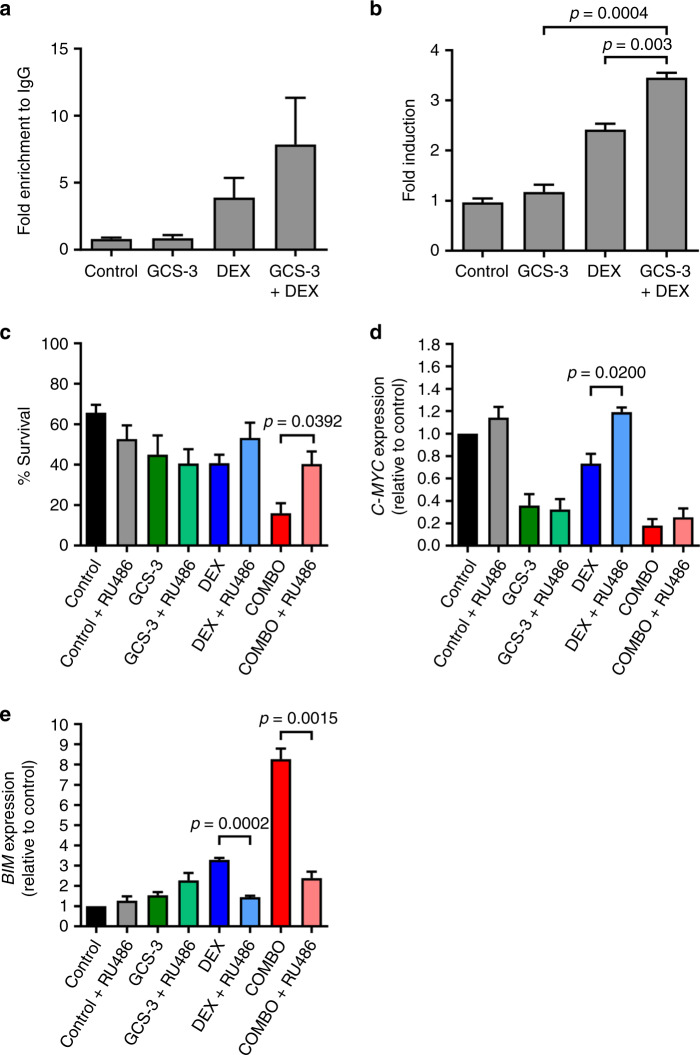
BIM upregulation correlates with GCS-3/dexamethasone efficacy. **a** ALL-19 xenograft cells were treated with 10 µM dexamethasone, 10 µM GCS-3 or both in combination for 8 h. Conventional ChIP of GR binding at the BIM-IGR, fold change was calculated relative to the IgG control. **b** Nalm6 cells that were stably transduced with a BIM promoter-reporter construct were treated with 1 µM dexamethasone, 10 µM GCS-3 or both in combination for 16 h. Fold change in luciferase induction was calculated. **c**–**e** ALL-19 cells were pre-treated with 1 µM RU486, then treated with 10 µM GSC-3, 10 µM dexamethasone and the combination for 24 h (qRT-PCR) or 48 h (cell sensitivity). **c** Cell sensitivity was assessed by flow cytometry. **d** *C-MYC* and **e** *BIM* mRNA expression was analysed by qRT-PCR and calculated relative to the control. Each data point represents the mean ± SEM of three independent experiments. Significance was calculated using the unpaired *t* test with Welch’s correction.

The activated GR can also trigger *BIM* expression by binding to a *BIM*-IGR, leading to BAX-mediated apoptosis. Conventional ChIP analysis showed that the GCS-3/dexamethasone combination treatment increased binding of the GR at the *BIM*-IGR by 9.2-fold compared to GCS-3 alone and 2.0-fold compared to dexamethasone alone (Fig. 6a). The GCS-3/dexamethasone combination also increased binding of the GR to *GILZ*, which is a primary target of the GR but is not directly involved in apoptosis (Supplementary Fig. S14). When the Nalm6 cell line was stably transduced with a *BIM* promoter-reporter construct,^20^ the GCS-3/dexamethasone combination significantly increased luciferase expression when compared to either single agent alone (Fig. 6b). In summary, GCS-3 enhanced dexamethasone-induced binding of the GR to the *BIM*-IGR, which significantly increased *BIM* promoter activity.

### *BIM* upregulation correlates with GCS-3/dexamethasone efficacy

Upon dexamethasone treatment the GR antagonist, RU486, allows GR translocation to the nucleus but inhibits *C-MYC* suppression.^33^ To determine whether *C-MYC* repression was critical for GCS-3/dexamethasone combination efficacy, the effect of RU486 on the combination treatment was assessed. Pre-incubation with RU486 increased the viability of ALL-19 cells treated with dexamethasone alone, and significantly abrogated the cytotoxic effects of the GCS-3/dexamethasone combination (Fig. 6c). Therefore, the cytotoxic effects of dexamethasone and the GCS-3/dexamethasone combination on ALL-19 cells are GR dependent.

While pre-incubation with RU486 had no effect on *C-MYC* expression of the control, GCS-3 and combination treated ALL-19 cells, it did significantly increase *C-MYC* expression of the dexamethasone-treated ALL-19 cells (Fig. 6d). Therefore, dexamethasone and GCS-3 downregulate *C-MYC* via GR-dependent and GR-independent pathways, respectively. Finally, GCS-3 as a single agent had no effect on *BIM* expression, but in combination with dexamethasone caused a significant increase (Fig. 6e). Notably, RU486 significantly suppressed the marked induction of *BIM* following treatment with the GCS-3/dexamethasone combination (Fig. 6e), consistent with its ability to reverse the cytotoxic effects of the combination (Fig. 6c). This was also observed with the dexamethasone-sensitive xenograft, ALL-54 (Supplementary Fig. S15). In summary, RU486 inhibited GCS-3/dexamethasone combination cytotoxicity and *BIM* upregulation with no effect on *C-MYC* expression, indicating that only *BIM* upregulation is required for the cytotoxicity of the GCS-3/dexamethasone combination against ALL-19 and ALL-54 cells.

## Discussion

Many drug classes have shown the potential to reverse glucocorticoid resistance in ALL, including histone deacetylase (HDAC), PI3K/AKT, mTOR, Pan-BCL-2, NOTCH, glycolysis, and cholesterol inhibitors.^19,34–47^ With the exception of J9, which was identified from an HTS assay against the T-ALL cell line, CUTLL1, none of these drugs were specifically developed as glucocorticoid sensitisers.^48^ We have previously tested J9 in a panel of dexamethasone-resistant and dexamethasone-sensitive ALL xenografts and observed no dexamethasone-sensitising effect.^26^ These conflicting results are likely to be due to the experimental models employed as ALL xenografts are not passaged in vitro and harvested cells remain highly representative of the cell and molecular characteristics of the primary patient sample.^24,25,49^

The novel glucocorticoid sensitiser, GCS-3, was identified from an unbiased HTS campaign against the B-ALL xenograft, ALL-19.^23,26^ GCS-3 was synergistic with dexamethasone in 7/20 ALL xenografts, which included both B-, T-, ETP- and Ph^+^ ALL subtypes and glucocorticoid-resistant and glucocorticoid-sensitive ALL xenografts. This was an impressive finding as a number of glucocorticoid sensitisers are specific to a particular subtype of ALL, such as the γ-secretase inhibitor DBZ, which specifically sensitises glucocorticoid-resistant T-ALL with activated NOTCH1 mutations.^44^ Glucocorticoid use is associated with a number of side effects such as infection, osteonecrosis, psychosis and myopathy.^50–54^ Therefore, if GCS-3 is able to potentiate the effects of glucocorticoids in glucocorticoid-sensitive ALL, as seen with ALL-54, lower doses of glucocorticoids may be administered, which will reduce side effects.

As a prelude to testing the GCS-3/dexamethasone combination in vivo, the maximum-tolerated dose of GCS-3 was determined in naive NSG mice. A pharmacokinetic study in naive NSG mice revealed that there was insufficient GCS-3 in the blood plasma to warrant further investigation in vivo. Although GCS-3 requires further development prior to in vivo testing, it is promising that synergy was not observed with GCS-3/dexamethasone in normal, human cells. While the precise mechanism of GCS-3 action requires further delineation, this study presents several interesting findings. GCS-3 is a specific glucocorticoid sensitiser with no sensitising effect observed when combined with vincristine, daunorubicin or cisplatin. While most glucocorticoid sensitisers are also glucocorticoid specific, Obatoclax has been shown to resensitise drug-resistant primary ALL cells to daunorubicin, vincristine and cytarabine, as well as dexamethasone.^43^ GCS-3 requires a functional GR to sensitise cells to glucocorticoids, but when combined with dexamethasone did not increase *GR* expression. Therefore, the mechanism of GCS-3 is different to the glucocorticoid sensitiser J9, which reverses dexamethasone resistance by upregulating *GR* expression.^48^

A somewhat surprising finding was that GCS-3 exerted maximal sensitising effect when administered simultaneously with dexamethasone. It is therefore unlikely that GCS-3 is an epigenetic modifier, as pre-treatment would be expected to enhance efficacy with dexamethasone, as observed with the HDAC inhibitor vorinostat.^19^ Microarray analysis of gene expression identified *C-MYC* as one of the most significantly downregulated genes between the control and combination treated groups, and the dexamethasone- and combination treated groups. This was confirmed by PCR and immunoblotting, which showed that the GCS-3/dexamethasone combination caused a significant decrease in *C-MYC* expression. This is a notable finding as multiple studies have shown that glucocorticoid treatment in ALL represses *C-MYC* expression, and glucocorticoid resistance is associated with *C-MYC* over-expression.^22,55–58^

Another promising finding from this study was that the GCS-3/dexamethasone combination significantly upregulated the expression of proapoptotic gene *BIM* compared to both single agents, which was confirmed by PCR and immunoblotting. Gene expression studies consistently show that *BIM* is upregulated by glucocorticoids in glucocorticoid-sensitive ALL samples.^55,56,59–61^ Our laboratory has shown that glucocorticoid resistance in ALL was associated with failure to induce *BIM* upon glucocorticoid treatment.^17,18^ Therefore, the combination of GCS-3 and dexamethasone induces gene expression changes in glucocorticoid-resistant ALL-19 cells, which are observed upon glucocorticoid treatment in glucocorticoid-sensitive ALL cells.

Previous work in our laboratory has identified two novel signalling pathways involved in glucocorticoid-induced apoptosis in ALL cells, and both centre on the opposing regulation of the proapoptotic gene, *BIM*, and the anti-apoptotic gene, *BCL-2*.^20^ The GCS-3/dexamethasone combination was able to enhance both *KLF13*-mediated *BCL-2* repression and increase GR binding to the *BIM*-IGR, which directly triggers *BIM* expression.

There are several studies that link *C-MYC* and *BIM* expression. A study performed in T-ALL cell lines and treatment-resistant patient samples showed that *BIM* is repressed downstream of *C-MYC* activation, and that *BIM* upregulation and apoptosis could be restored by treatment with C-MYC or PI3K-AKT pathway inhibitors.^62^ A study performed in the colon cancer cell line, HCT116, showed that genes encoding the BH3-only proteins *NOXA* and *BIM* are direct targets of *C-MYC*.^63^ Interestingly, that study showed that *C-MYC* acts in a complex with the transcription factor, early growth response 1 (EGR1), which was the fourth most downregulated gene between dexamethasone- and combination treated groups in our study. A study performed in the T-ALL cell line, Jurkat, showed that EGR1 binds and transactivates the *BIM* locus.^64^ However, in our microarray study *EGR1* was strongly downregulated by the combination treatment and *BIM* was upregulated. In our study, GCS-3/dexamethasone-induced downregulation of *C-MYC* was not inhibited by the GR antagonist, RU486; however, the efficacy of the combination was inhibited. GCS-3/dexamethasone-induced upregulation of *BIM* was inhibited by RU486; therefore, *BIM* upregulation correlates with GCS-3/dexamethasone combination efficacy and *BIM* does not appear to be a target of *C-MYC* in ALL-19 or ALL-54.

While it is important to develop new chemotherapeutics for the treatment of paediatric ALL, it is also useful to revisit and reinvigorate chemotherapeutic agents, such as glucocorticoids, which have a proven track record in paediatric ALL treatment. Reversing glucocorticoid resistance in paediatric ALL has the potential to improve the outcome for newly diagnosed high-risk patients with intrinsic glucocorticoid resistance, and patients with relapsed ALL and acquired resistance to glucocorticoids. Further development of GCS-3 may lead to a new class of drugs for the pharmacological reversal of glucocorticoid resistance in paediatric ALL, and ultimately to improved outcomes for patients with intrinsic and acquired glucocorticoid-resistant-lymphoid malignancies.

## Supplementary information

Supplementary

## Data Availability

All data generated or analysed during this study are included in this manuscript. Supplementary information is available at the *British Journal of Cancer* website.
