# Supplementary material for: Reversal of glucocorticoid resistance in paediatric acute lymphoblastic leukaemia is dependent on restoring BIM expression
Source: Br J Cancer. 2020 Apr 3;122(12):1769–81. doi: 10.1038/s41416-020-0824-8 (PMC7283241; doi:10.1038/s41416-020-0824-8)
Supplement: Supplementary file 1 — Supplementary [file 41416_2020_824_MOESM1_ESM.pdf]

Reversal of glucocorticoid resistance in paediatric acute lymphoblastic leukaemia is dependent on restoring *BIM* expression

Cara E Toscan,[1] Duohui Jing,[1] Chelsea Mayoh[1] and Richard B Lock[1]

[1]Children's Cancer Institute, School of Women's and Children's Health, UNSW Sydney, Australia

SUPPLEMENTAL MATERIALS

SUPPLEMENTARY METHODS

Reagents and equipment for cell culture and cytotoxicity assays

Cell culture tested, water-insoluble dexamethasone and prednisolone were used in all experiments (Sigma, NSW, Australia). QVD-OPh and RU38486 were also purchased from Sigma. Vincristine and cisplatin were purchased from The Prince of Wales Hospital Pharmacy (NSW, Australia). Daunorubicin was purchased from Steritech Pty Ltd (NSW, Australia). GCS-3 was purchased from Reagency Pty Ltd (VIC, Australia). Flow cytometry was performed using a Guava easyCyte flow cytometer (Merck, NSW, Australia) or a BD FACS Canto (BD Biosciences, NSW, Australia). Data acquired using flow cytometry was analysed using FacsDiva software (BD Biosciences). Cellular viability determined by Resazurin reagent was measured using a fluorimeter (Molecular Devices, San Jose, CA), with excitation at 530 nm and emission at 590 nm.

SUPPLEMENTARY TABLES

Supplementary Table S1. Patient demographics of ALL xenografts and *ex vivo* response to dexamethasone. The sensitivity of ALL xenograft cells to dexamethasone (DEX) *ex vivo* was assessed by Resazurin cytotoxicity assay following 48 h drug exposure.

Xenograft	ALL subtype	Key genomic lesions	Disease status at biopsy	Current clinical status	*Ex vivo* DEX IC_{50} (µM)
ALL-19	B-ALL	NUP214-ABL1	Relapse	DOD	> 40
ALL-2	B-ALL	-	Relapse	DOD	> 40
ALL-50	B-ALL	CRLF2-CLVS1	Diagnosis	DOD	> 40
ALL-7	B-ALL	TCF3-HLF	Diagnosis	DOD	> 40
ALL-88	B-ALL	PAX5-JAK2	Relapse	DOD	> 40
ALL-84	B-ALL	-	Relapse	DOD	> 40
ALL-54	B-ALL	PAX5-NBPF8	Diagnosis	CR1	0.060
ALL-17	B-ALL	-	Diagnosis	CR2	0.036
ALL-4	Ph+-ALL	BCR-ABL1	Diagnosis	DOD	> 40
ALL-55	Ph+-ALL	BCR-ABL1	Diagnosis	CR1	0.093
ALL-56	Ph+-ALL	BCR-ABL1	Diagnosis	CR3	0.016
MLL-5	Infant *MLL*r-ALL	KMT2A-MLLT10	Diagnosis	-	> 40
ALL-3	Non-infant *MLL*r-ALL	KMT2A-MLLT1	Diagnosis	CR2	0.012
ALL-31	T-ALL	-	Diagnosis	DOD	> 40
ALL-32	T-ALL	NUP214-ABL1	Relapse	CR2	> 40
ALL-43	T-ALL	-	Diagnosis	CR2	0.021
ALL-16	T-ALL	-	Diagnosis	CR1	0.004
ETP-2	ETP-ALL	-	Diagnosis	CR1	> 40
ETP-3	ETP-ALL	-	Diagnosis	DOD	> 40
ETP-1	ETP-ALL	-	Diagnosis	DOD	0.041

*MLL*r, *mixed-lineage leukaemia*-rearranged; Ph^+, Philadelphia chromosome positive ALL; ETP, early T-cell precursor ALL; DOD, dead of disease; CR1/2/3, alive in first/second/third complete remission.

Supplementary Table S2. ***Ex vivo*** **efficacy of GCS-3 in combination with dexamethasone or prednisolone against ALL-19 xenograft cells.** ALL-19 xenograft cells were exposed to GCS-3, glucocorticoid, or both in combination at a 1:1 fixed-ratio of concentrations for 48 h, or 12 h followed by drug removal and fresh media for 36 h. Cell viability was assessed by Resazurin assay or flow cytometry. Deviation from Bliss-additivity (BA) was calculated at each tested concentration.

**Cell viability assessment**	**Glucocorticoid**	**Deviation from BA at each tested ratio;**						
		**0.25**	**0.5**	**1.0**	**2.0**	**4.0**	**Average**	**Comb. Effect**
Resazurin assay	dexamethasone	0.00	0.00	0.06	0.16	0.02	0.05	Synergism
Resazurin assay	prednisolone	0.08	0.11	0.23	0.34	0.09	0.17	Synergism
Flow cytometry	dexamethasone	-0.04	0.04	0.04	0.07	0.11	0.04	Nearly Additive
Resazurin assay	dexamethasone 12h, then removal	-0.02	-0.07	-0.01	-0.03	0.05	-0.01	Nearly Additive

Supplementary Table S3. ***Ex vivo*** **efficacy of GCS-3 in combination with dexamethasone against non-leukemic, human cells.** Human PBMCs and CD34+ cells were exposed to GCS-3, dexamethasone, or both in combination at a 1:1 fixed-ratio of concentrations for 48 h. Cell sensitivity was assessed by Resazurin cytotoxicity assay. Deviation from Bliss-additivity (BA) was calculated at each tested concentration.

Sample	Cell type	Deviation from BA at each tested ratio;						
		0.25	0.5	1.0	2.0	4.0	Average	Comb. Effect
PBMC 1	PBMC	-0.07	-0.06	-0.05	0.03	0.04	-0.02	Nearly Additive
PBMC 2	PBMC	-0.07	-0.04	0.00	0.11	0.11	0.02	Nearly Additive
PBMC 3	PBMC	-0.01	-0.01	0.03	0.06	0.11	0.04	Nearly Additive
CD34+ 1	CD34+	0.06	0.02	-0.01	-0.04	-0.07	-0.01	Nearly Additive
CD34+ 2	CD34+	0.04	-0.01	0.01	-0.05	-0.08	-0.02	Nearly Additive
CD34+ 3	CD34+	-0.01	-0.07	-0.03	-0.07	-0.09	-0.05	Antagonism

Supplementary Table S4. ***Ex vivo*** **efficacy of GCS-3 in combination with non-glucocorticoids against ALL-19 xenograft cells.** ALL-19 xenograft cells were exposed to a GCS-3, non-glucocorticoid, or both in combination at a fixed-ratio of concentrations for 48 h. Cell sensitivity was then assessed by Resazurin cytotoxicty assay. The ratio of GCS-3 to drug (G/D ratio) is determined from single agent assays. Deviation from Bliss-additivity (BA) was calculated at each tested concentration.

**Non-glucocorticoid**	**Xenograft**	**G/D ratio**	**Deviation from BA at each tested ratio;**						
			**0.25**	**0.5**	**1.0**	**2.0**	**4.0**	**Average**	**Comb. Effect**
Daunorubicin	ALL-19	5000:41	-0.03	-0.09	-0.14	-0.09	-0.05	-0.08	Antagonism
Vincristine	ALL-19	10000:23	-0.03	-0.03	-0.09	-0.02	-0.03	-0.04	Nearly Additive
Cisplatin	ALL-19	1:1	-0.07	-0.11	-0.13	-0.02	-0.09	-0.08	Antagonism

Supplementary Table S5. *Ex vivo* efficacy of GCS-3 in combination with dexamethasone against ALL xenograft cells. Xenograft cells were exposed to GCS-3, dexamethasone (DEX), or both in combination at a fixed-ratio of concentrations for 48 h. Cell sensitivity was then assessed by Resazurin cytotoxicty assay. The ratio of GCS-3 to dexamaethasone (G/D ratio) is determined from single agent assays. Deviation from Bliss-additivity (BA) was calculated at each tested concentration.

**Xenograft**	**ALL Subtype**	***Ex vivo* DEX IC_{50} (μM)**	**G/D ratio**	**Deviation from BA at each tested ratio; 0.25**	**0.5**	**1.0**	**2.0**	**4.0**	**Average**	**Comb. Effect**
ALL-2	B-ALL	> 40	1:1	-0.06	-0.05	0.00	0.05	-0.03	-0.02	Nearly Additive
ALL-50	B-ALL	> 40	1:1	-0.01	-0.01	-0.01	0.12	0.05	0.03	Nearly Additive
ALL-7	B-ALL	> 40	1:1	0.00	-0.04	-0.03	0.31	0.66	0.18	Synergism
ALL-88	B-ALL	> 40	1:1	-0.08	-0.02	0.18	0.15	0.00	0.04	Nearly Additive
ALL-84	B-ALL	> 40	1:1	0.04	0.13	0.25	0.38	0.23	0.21	Synergism
ALL-54	B-ALL	0.060	5000:3	0.01	0.06	0.23	0.23	0.07	0.12	Synergism
ALL-17	B-ALL	0.036	2500:9	-0.09	-0.01	0.00	-0.06	-0.08	-0.05	Antagonism
ALL-4	Ph^+-ALL	> 40	1:1	-0.01	0.02	0.13	0.22	0.07	0.09	Synergism
ALL-55	Ph^+-ALL	0.093	10000:93	-0.05	0.03	0.12	0.01	-0.02	0.02	Nearly Additive
ALL-56	Ph^+-ALL	0.016	625:1	0.03	0.10	0.02	0.04	-0.02	0.03	Nearly Additive
MLL-5	*MLL*r-ALL	> 40	1:1	0.00	0.00	-0.08	-0.02	0.00	-0.02	Nearly Additive
ALL-3	*MLL*r-ALL	0.012	2500:3	-0.06	-0.09	-0.15	-0.09	-0.04	-0.09	Antagonism
ALL-31	T-ALL	> 40	1:1	-0.01	-0.01	0.01	0.16	0.29	0.09	Synergism
ALL-32	T-ALL	> 40	1:1	-0.04	-0.05	-0.04	-0.03	-0.05	-0.04	Nearly Additive
ALL-43	T-ALL	0.021	10000:21	-0.04	-0.08	-0.04	-0.06	-0.15	-0.07	Antagonism
ALL-16	T-ALL	0.004	2500:1	-0.12	-0.19	-0.19	-0.08	-0.04	-0.12	Antagonism
ETP-2	ETP-ALL	> 40	1:1	-0.03	-0.05	-0.05	0.08	0.29	0.05	Synergism
ETP-3	ETP-ALL	> 40	1:1	-0.01	-0.05	-0.01	0.11	0.04	0.02	Nearly Additive
ETP-1	ETP-ALL	0.041	10000:41	-0.03	-0.07	-0.05	0.04	-0.05	-0.03	Nearly Additive

*MLL*r, *mixed-lineage leukaemia*-rearranged; Ph+, Philadelphia chromosome positive ALL; ETP, early T-cell precursor ALL.

Supplementary Table S6. *In vitro* efficacy of GCS-3 in combination with dexamethasone against glucocorticoid resistant cell lines. HAL-01, UoC-B1 or ALL-4CL cells were exposed to a GCS-3, dexamethasone, or both in combination at a fixed-ratio of concentrations for 48 h. Cell sensitivity was then assessed by Resazurin cytotoxicty assay. The ratio of GCS-3 to drug (G/D ratio) is determined from single agent assays. Deviation from Bliss-additivity (BA) was calculated at each tested concentration.

Cell line	ALL Subtype	G/D ratio	Deviation from BA at each tested ratio;						
			**0.25**	**0.5**	**1.0**	**2.0**	**4.0**	**Average**	**Comb. Effect**
HAL-01	BCP-ALL	1:1	0.00	-0.05	-0.08	-0.04	-0.05	-0.04	Nearly Additive
UoC-B1	BCP-ALL	1:1	0.00	-0.01	0.01	-0.03	-0.01	-0.01	Nearly Additive
ALL-4CL	BCP-ALL	1:1	0.02	0.05	0.17	0.14	-0.02	0.07	Synergism

Supplementary Table S7. Number of differentially expressed genes between each treatment group. Differential gene expression was performed on 34,694 genes. The number of differentially expressed genes with a p-value < 0.05 and a fold-change (FC) ≥ |2| are shown for each time point

**Comparison**	**Number of differentially expressed genes**	
	**12 h**	**24 h**
control vs dexamethasone	389	471
control vs GCS-3	68	330
control vs combination	490	1049
GCS-3 vs combination	386	675
**dexamethasone vs combination**	**46**	**201**

SUPPLEMENTARY FIGURES

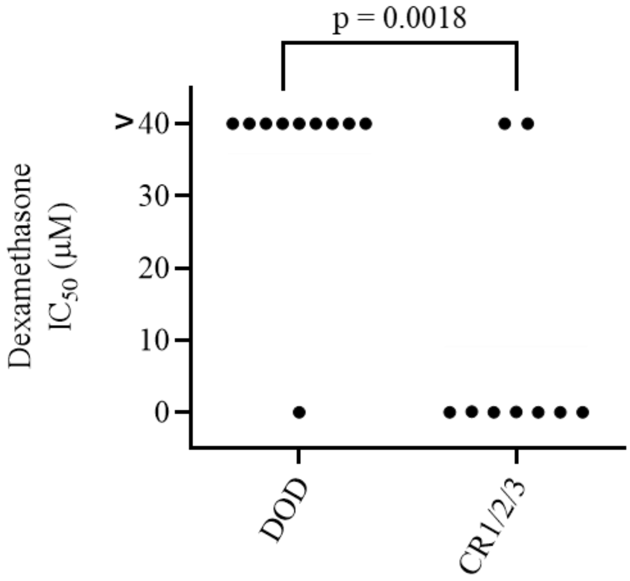

Supplementary Figure S1. Correlation between patient clinical status and *ex vivo* response of ALL xenografts to dexamethasone. The sensitivity of 19 ALL xenograft cells to dexamethasone *ex vivo* was assessed by Resazurin cytotoxicity assay following 48 h drug exposure. Stratification based on patient outcome, dead of disease (DOD) or alive in first/second/third complete remission (CR1/2/3). Significance calculated using unpaired t-test with Welch's correction.

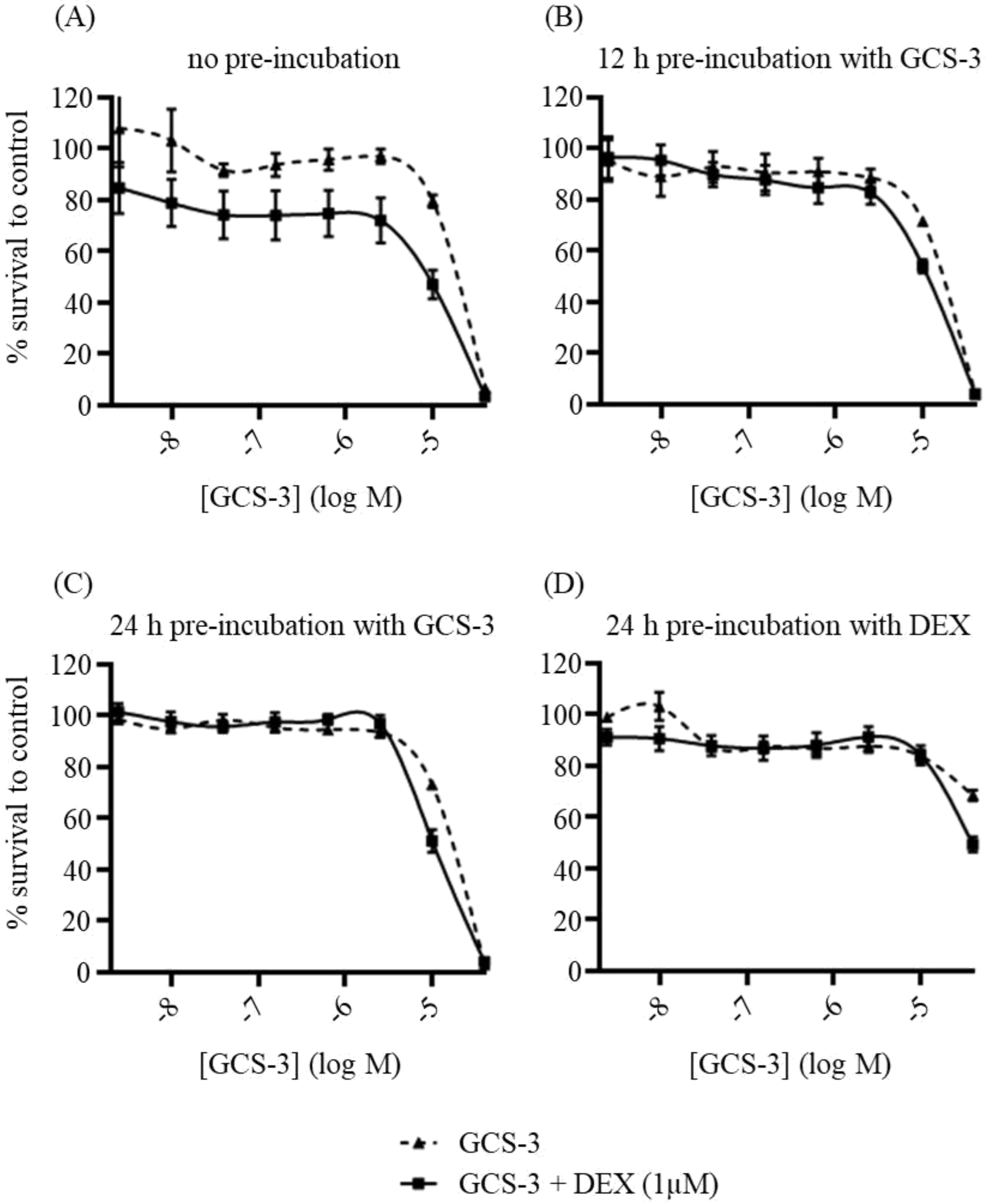

Supplementary Figure S2. Order of addition experiments with GCS-3 and dexamethasone against ALL-19. (A) ALL-19 cells were treated simultaneously with a dose-response of GCS-3 ± 1 μM dexamethasone for 48 h. (B) ALL-19 cells were pre-treated for 12 h with GCS-3 before the addition of ± 1 μM dexamethasone for 48 h. (C) ALL-19 cells were pre-treated for 24 h with GCS-3 before the addition of ± 1 μM dexamethasone for 48 h. (D) ALL-19 cells were pre-treated for 24 h with ± 1 μM dexamethasone before the addition of GCS-3 for 24 h. For each treatment cells were exposed to dexamethasone for 48 h. Cell sensitivity was assessed by Resazurin assay. Each data point represents the mean ± SEM of three independent experiments.

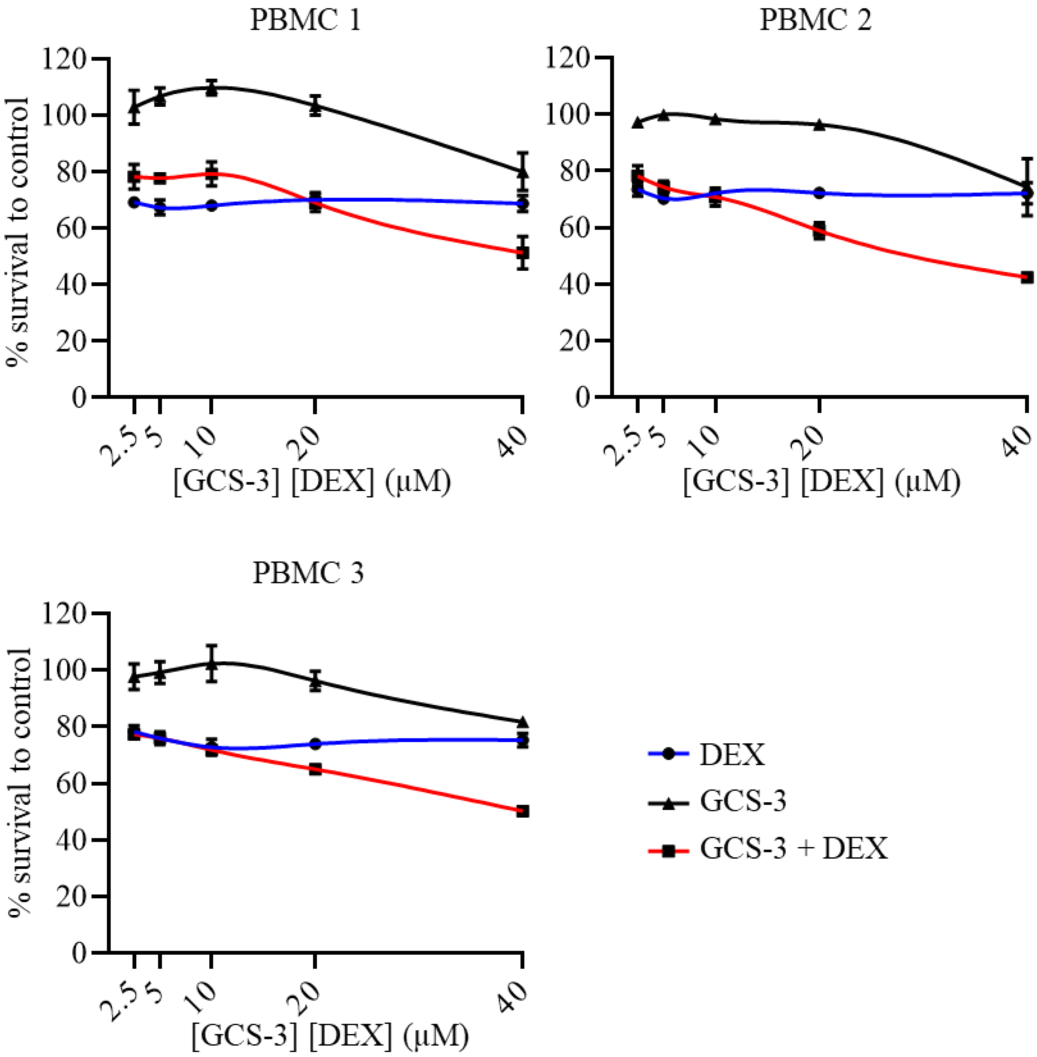

Supplementary Figure S3. *Ex vivo* efficacy of GCS-3 in combination with dexamethasone against human PBMC cells. Human PBMC cells from 3 separate donors were exposed to GCS-3, dexamethasone, or both in combination at a fixed-ratio of concentrations for 48 h, and cell sensitivity was then assessed by Resazurin cytotoxicity assay. Each data point represents the mean ± SEM of three independent experiments.

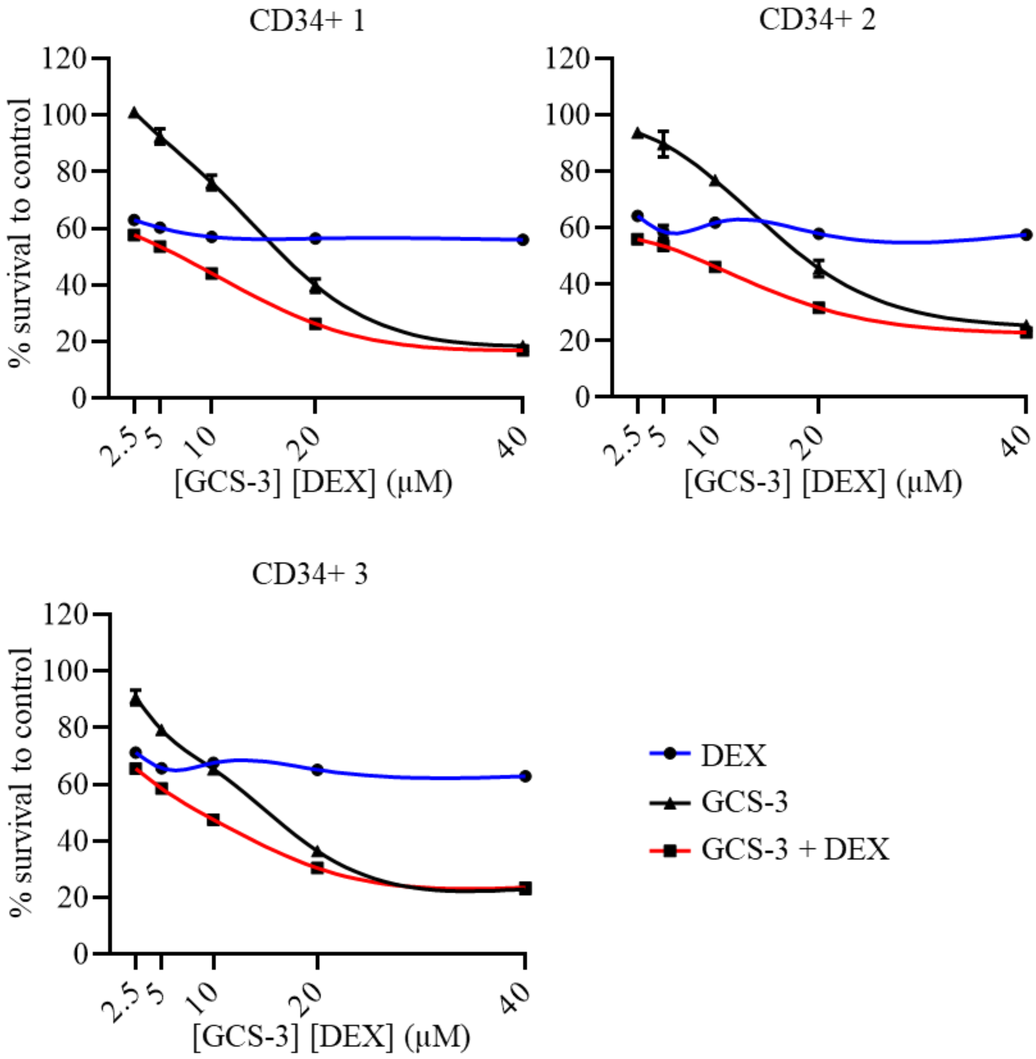

Supplementary Figure S4. *Ex vivo* efficacy of GCS-3 in combination with dexamethasone against human CD34+ cells. Human CD34+ cells from 3 separate donors were exposed to GCS-3, dexamethasone, or both in combination at a fixed-ratio of concentrations for 48 h, and cell sensitivity was then assessed by Resazurin cytotoxicity assay. Each data point represents the mean ± SD of one independent experiments.

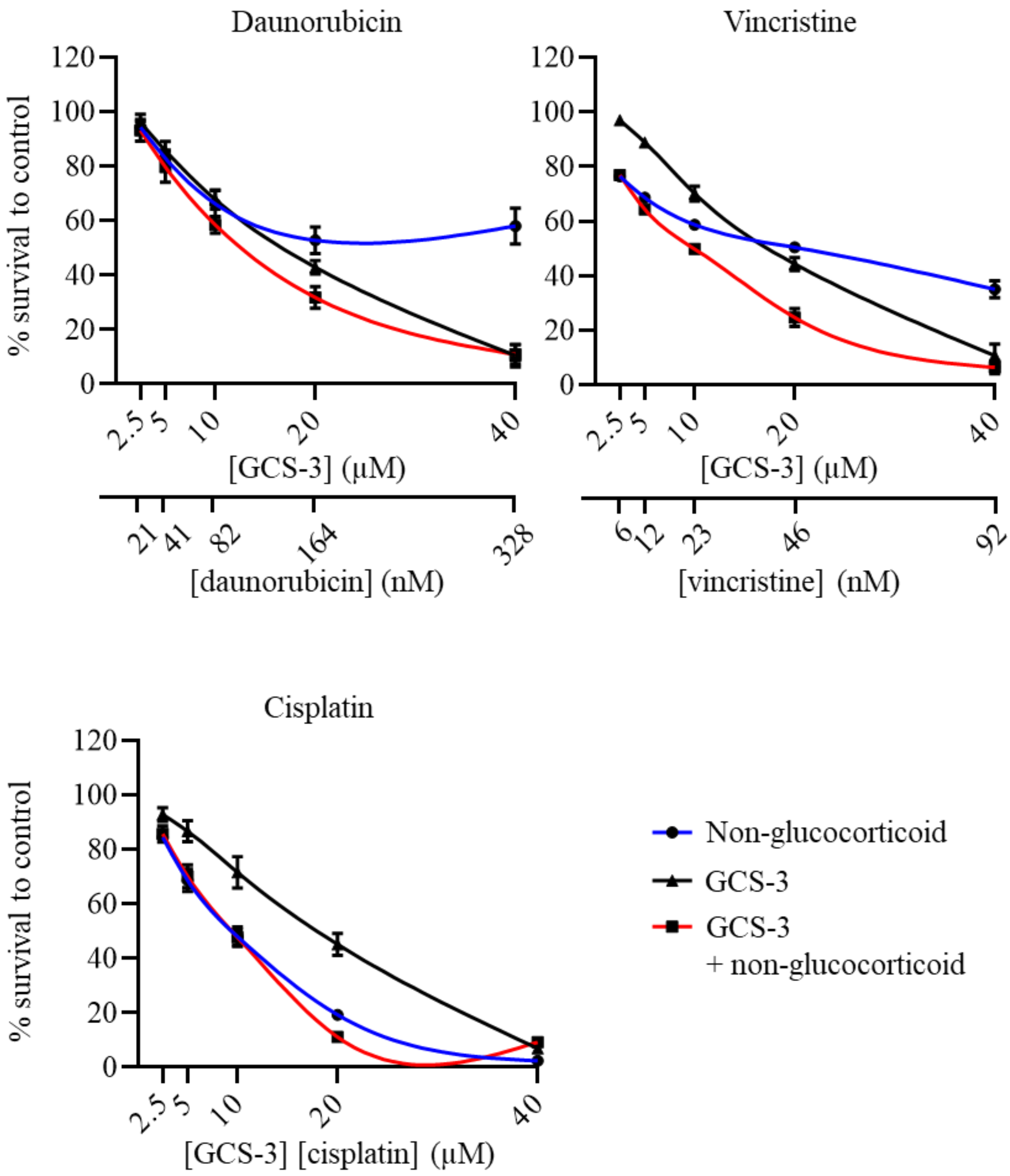

Supplementary Figure S5. *Ex vivo* efficacy of GCS-3 in combination with non-glucocorticoids against ALL-19 xenograft cells. ALL-19 xenograft cells were exposed to GCS-3, non-glucocorticoid, or both in combination at a fixed-ratio of concentrations for 48 h, and cell sensitivity was then assessed by Resazurin cytotoxicity assay. Each data point represents the mean ± SEM of at least three independent experiments.

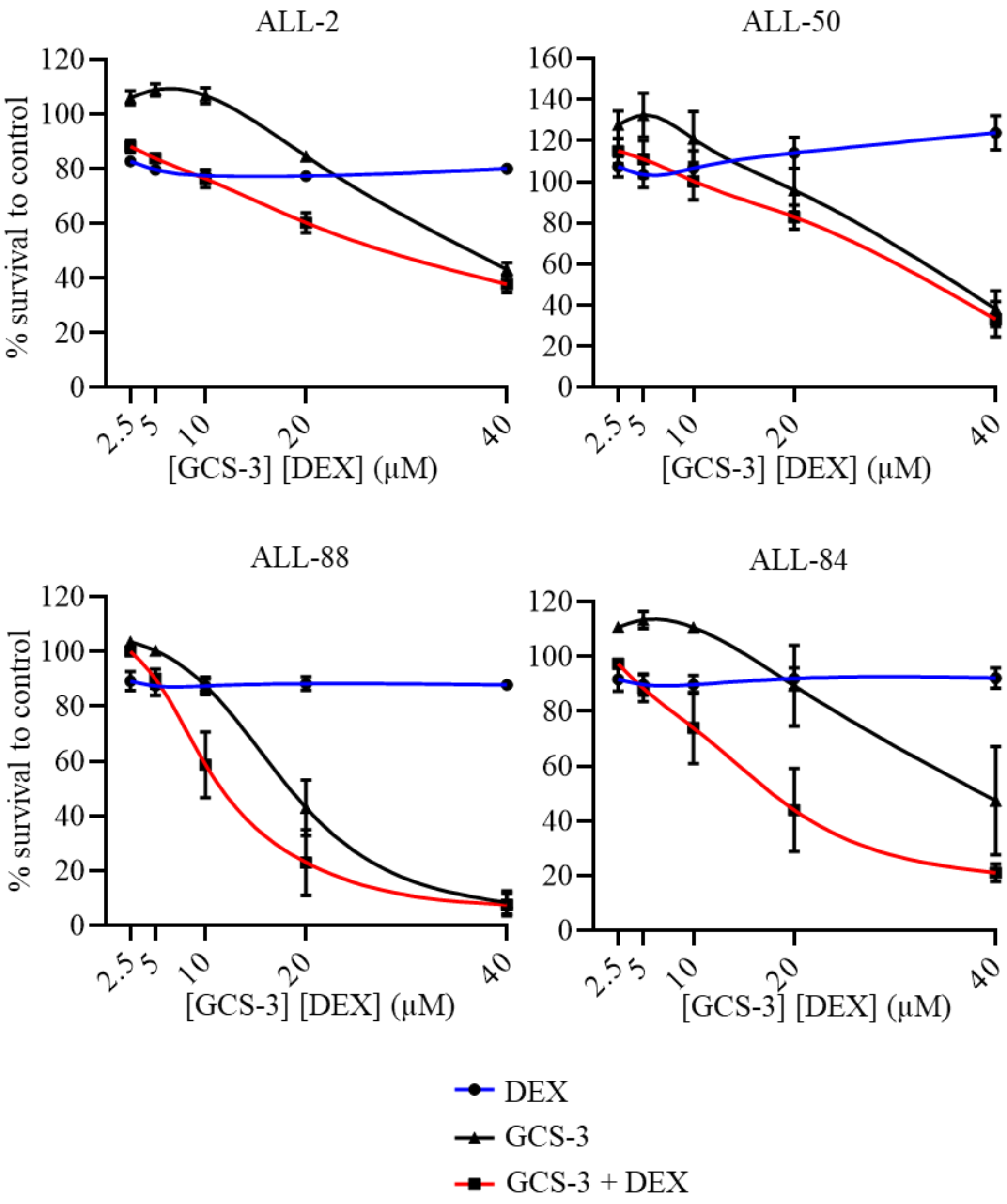

Supplementary Figure S6. ***Ex vivo*** **efficacy of GCS-3 in combination with dexamethasone against ALL xenograft cells.** ALL xenograft cells were exposed to GCS-3, dexamethasone, or both in combination at a fixed-ratio of concentrations for 48 h, and cell sensitivity was then assessed by Resazurin cytotoxicity assay. Each data point represents the mean ± SEM of at least three independent experiments.

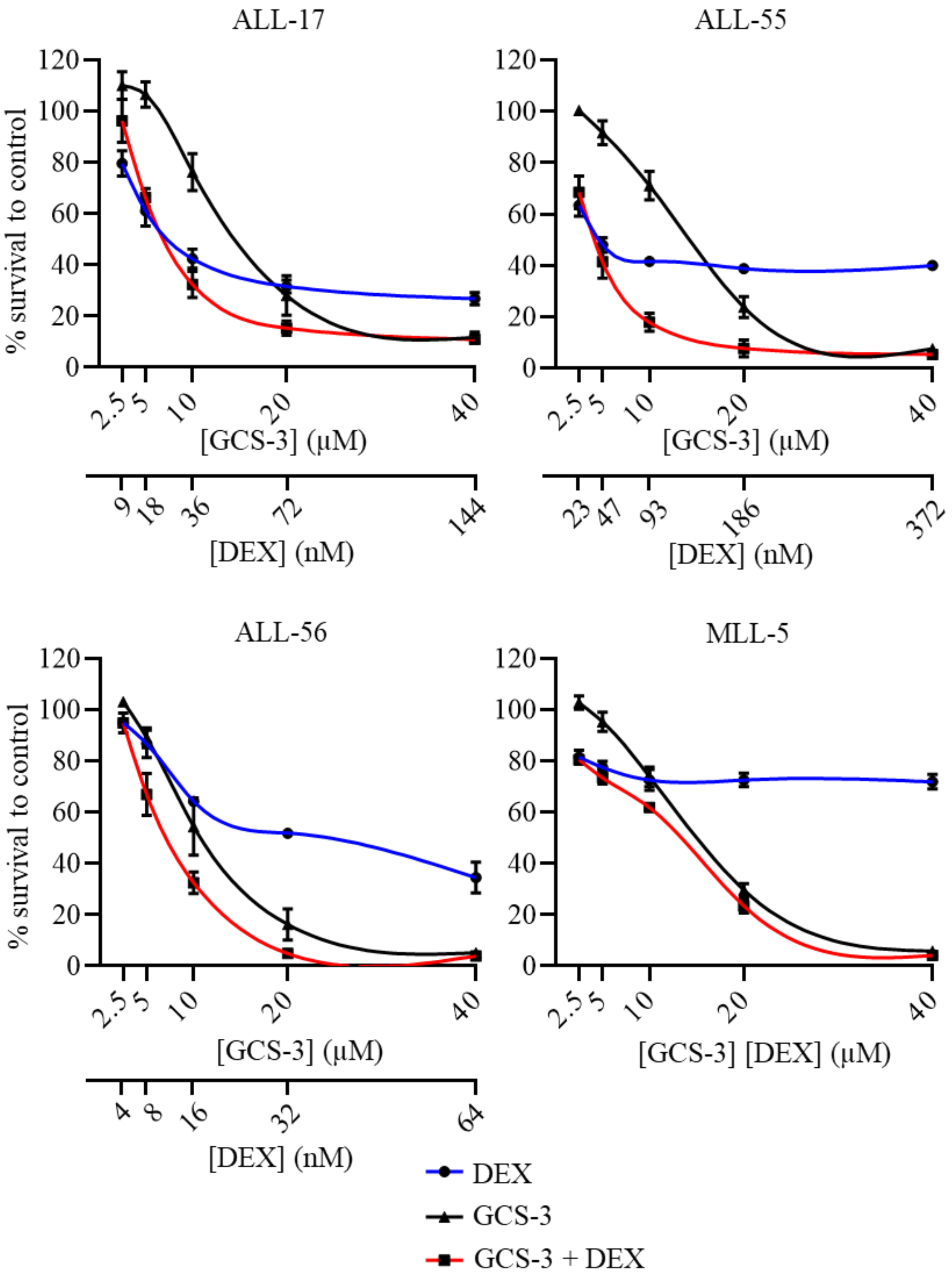

Supplementary Figure S6 continued. *Ex vivo* efficacy of GCS-3 in combination with dexamethasone against ALL xenograft cells. ALL xenograft cells were exposed to GCS-3, dexamethasone, or both in combination at a fixed-ratio of concentrations for 48 h, and cell sensitivity was then assessed by Resazurin cytotoxicity assay. Each data point represents the mean ± SEM of at least three independent experiments.

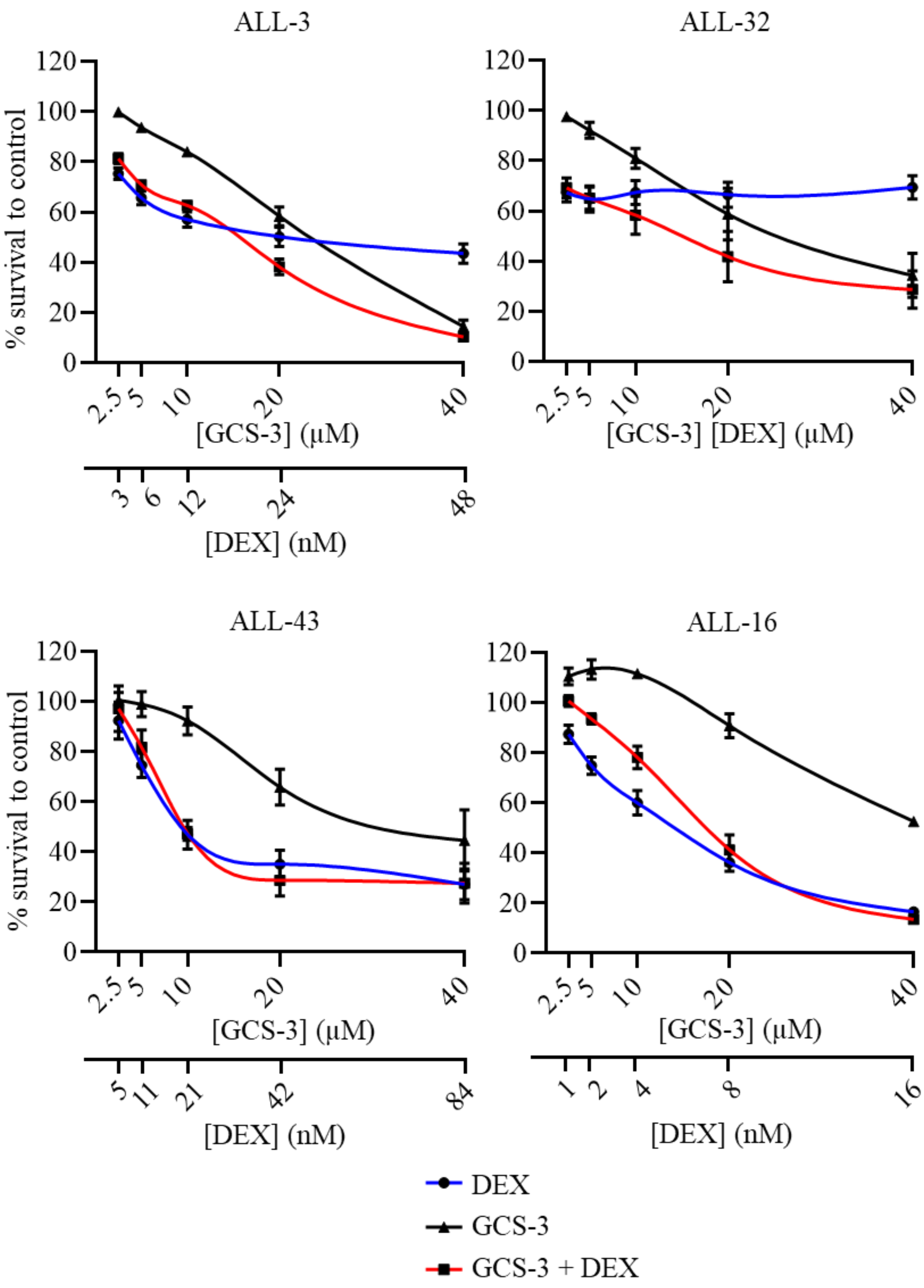

Supplementary Figure S6 continued. ***Ex vivo*** **efficacy of GCS-3 in combination with dexamethasone against ALL xenograft cells.** ALL xenograft cells were exposed to GCS-3, dexamethasone, or both in combination at a fixed-ratio of concentrations for 48 h, and cell sensitivity was then assessed by Resazurin cytotoxicity assay. Each data point represents the mean ± SEM of at least three independent experiments.

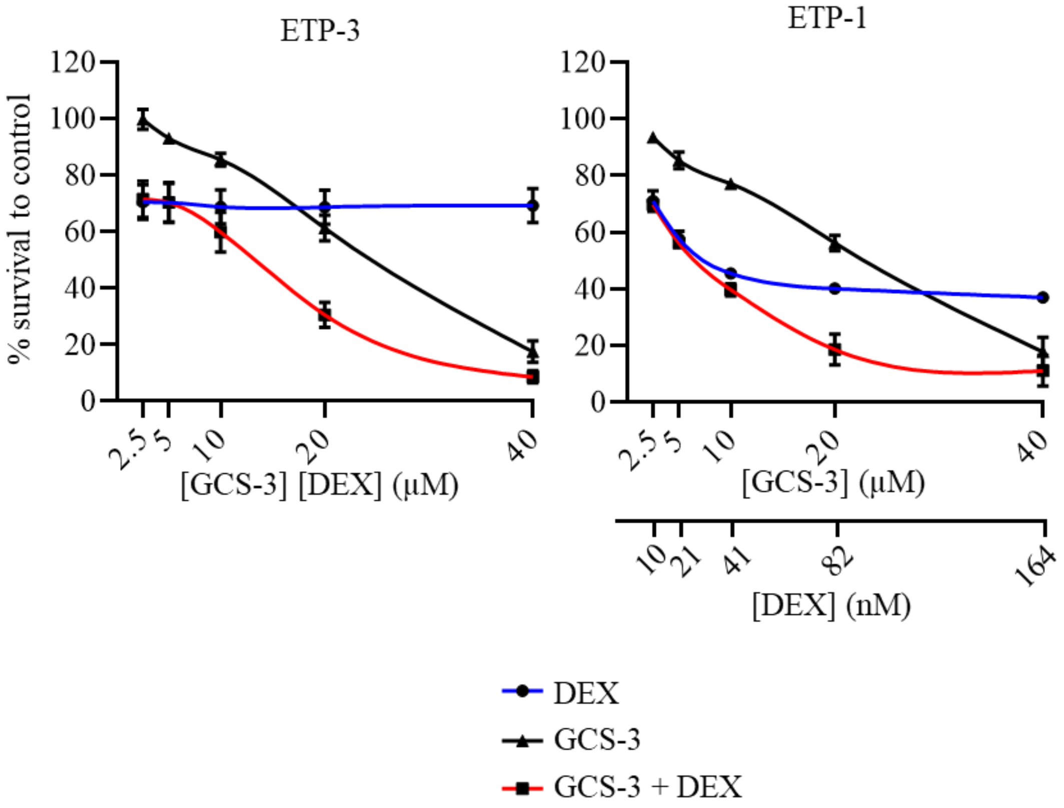

Supplementary Figure S6 continued. *Ex vivo* efficacy of GCS-3 in combination with dexamethasone against ALL xenograft cells. ALL xenograft cells were exposed to GCS-3, dexamethasone, or both in combination at a fixed-ratio of concentrations for 48 h, and cell sensitivity was then assessed by Resazurin cytotoxicity assay. Each data point represents the mean ± SEM of at least three independent experiments.

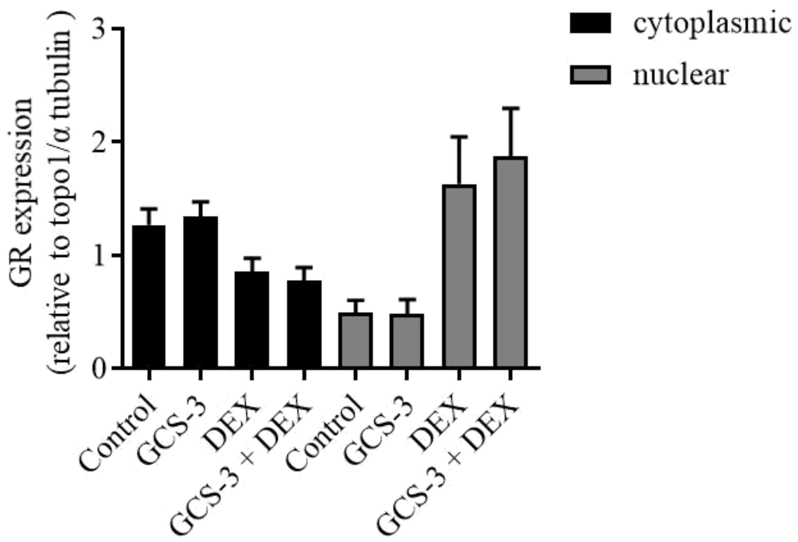

Supplementary Figure S7. Effect of GCS-3/dexamethasone on nuclear and cytoplasmic GR expression. ALL-19 xenograft cells were treated with 10 µM GCS-3, 1 µM dexamethasone (DEX) for 1 h before separation into nuclear and cytoplasmic fractions. Equal amounts of protein (10 µg) from each fraction were immunoblotted for GR, topoisomerase 1 (nuclear loading control) and α tubulin (cytoplasm loading control). GR expression relative to loading control is shown. Each data point represents the mean ± SEM of three independent experiments.

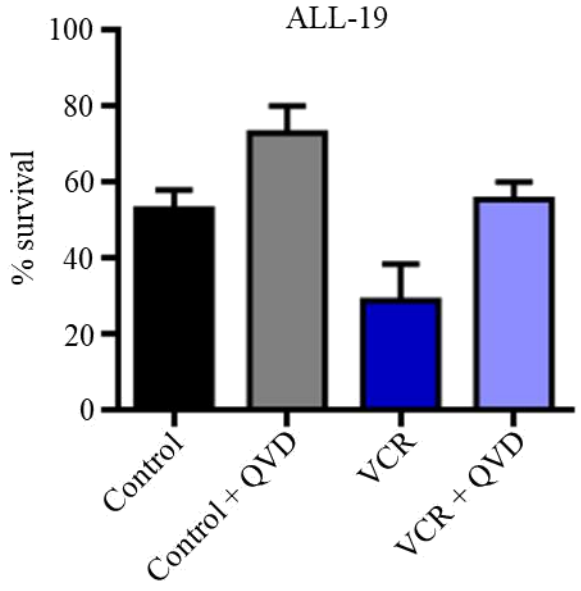

Supplementary Figure S8. Effect of the pan-caspase inhibitor, QVD-OPh, on response of ALL-19 cells to vincristine. ALL-19 xenograft cells were pre-treated with 10 µM QVD-OPh (QVD) or vehicle control for 2 h. Cells were then exposed to 23 nM of vincristine (VCR) for 48 h and cell sensitivity was assessed by flow cytometry. Each data point represents the mean ± SEM of four independent experiments.

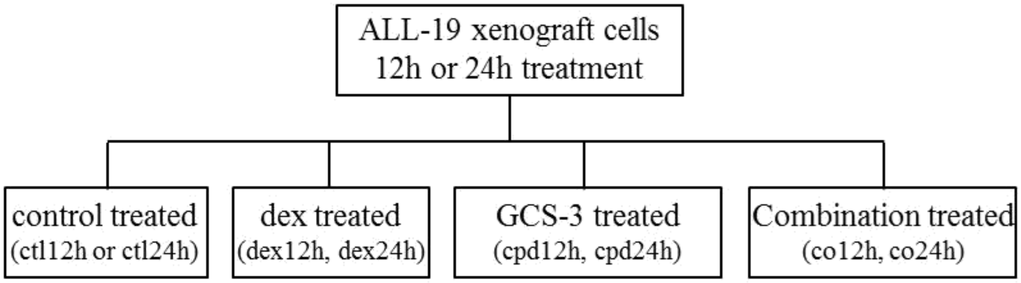

Supplementary Figure S9. Gene expression profiling treatment groups. ALL-19 xenograft cells were treated with 10 µM dexamethasone, 10 µM GCS-3, or both in combination for 12 or 24 h. Gene expression profiling was performed on each of the 8 treatment groups in duplicate.

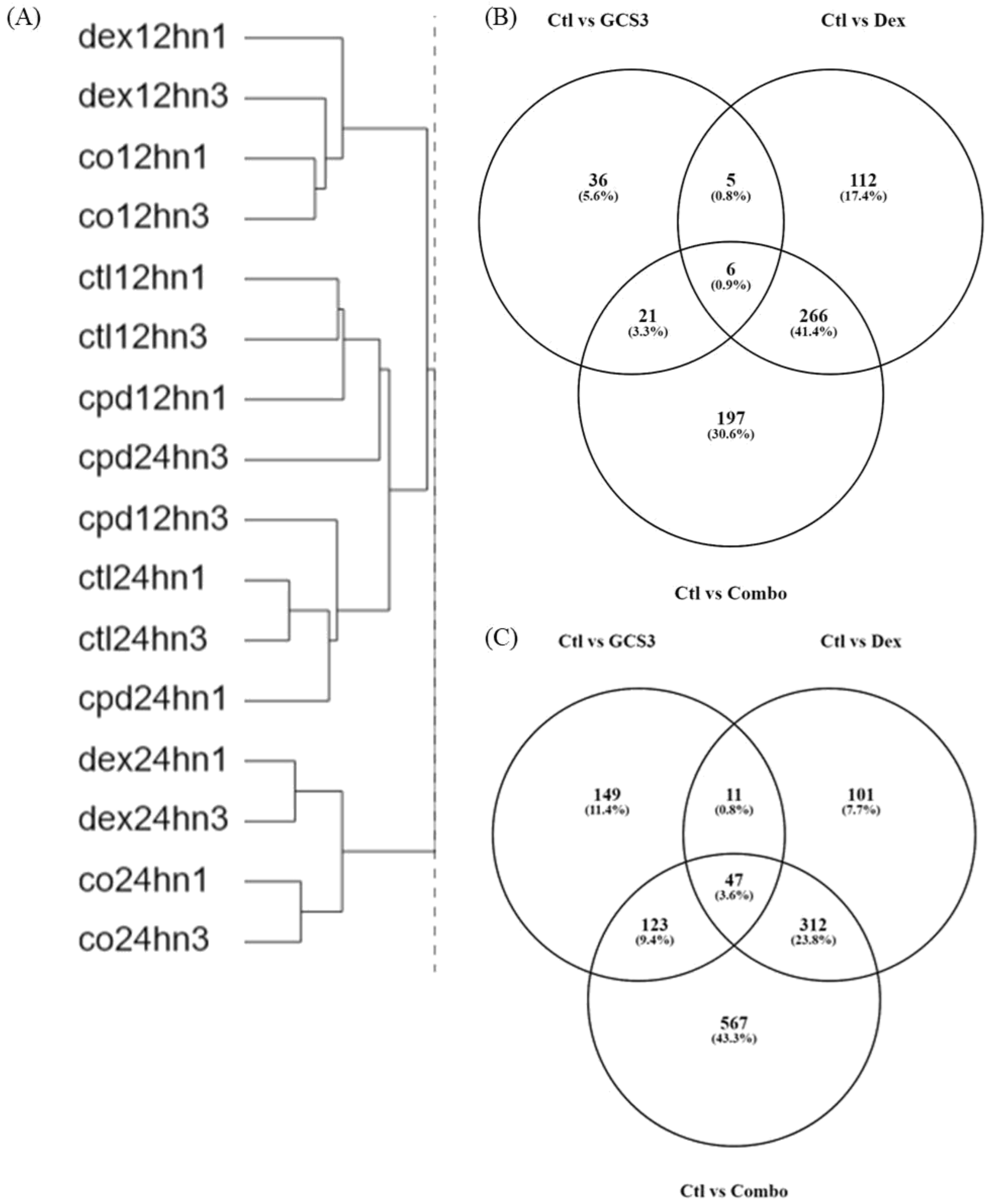

Supplementary Figure S10. Hierarchical clustering and differentially expressed genes. ALL-19 xenograft cells were treated with vehicle (ctl), 10 µM dexamethasone (dex), 10 µM GCS-3 (cpd), or both in combination (co) for 12 or 24 h, and gene expression profiling was performed. (A) Unsupervised hierarchical clustering was performed on the 16 samples using the Pairwise complete linkage clustering method. Venn diagrams of the number of differentially expressed genes between control vs GCS-3, control vs dexamethasone (Dex), and control vs combination (Combo) after 12 h (B) and 24 h (C) treatment.

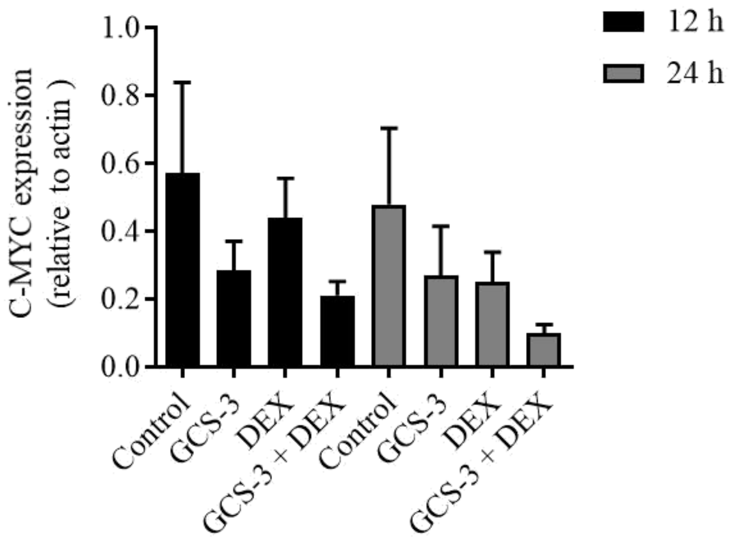

Supplementary Figure S11. Effect of GCS-3/dexamethasone on C-MYC expression. ALL-19 xenograft cells were treated with 10 µM dexamethasone (DEX), 10 µM GCS-3, or both in combination for 12 h and 24 h. Equal amounts of protein (20 µg) were immunoblotted for C-MYC and actin (loading control). C-MYC expression relative to loading control is shown. Each data point represents the mean ± SEM of three independent experiments.

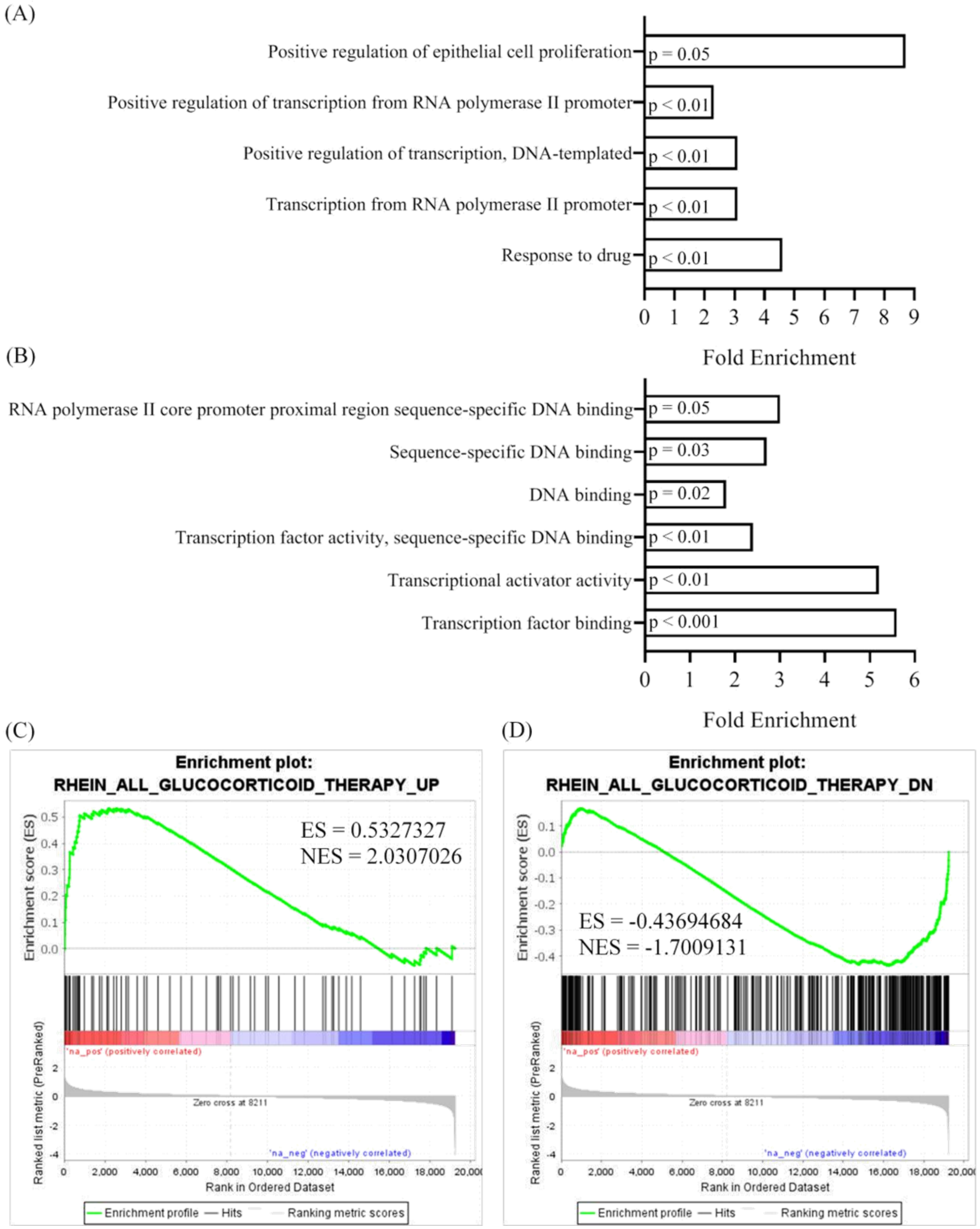

Supplementary Figure S12. GO and GSEA analysis of differentially expressed genes. (A-B) Gene Ontology (GO) analysis was performed on differentially expressed genes that were downregulated between the 24 h dexamethasone and 24 h combination groups. Key biological processes (A) and

molecular functions (B) involving *C-MYC* are shown. (C-D) Differentially expressed genes between the 24 h combination and 24 h dexamethasone treated groups were ranked, then GSEA was performed. GSEA plots of the Rhein ALL Glucocorticoid therapy up (C) and down (D) gene sets in the combination vs dexamethasone treated groups are shown. Enrichment scores (ES) and normalised enrichment scores (NES) are shown for each gene set.

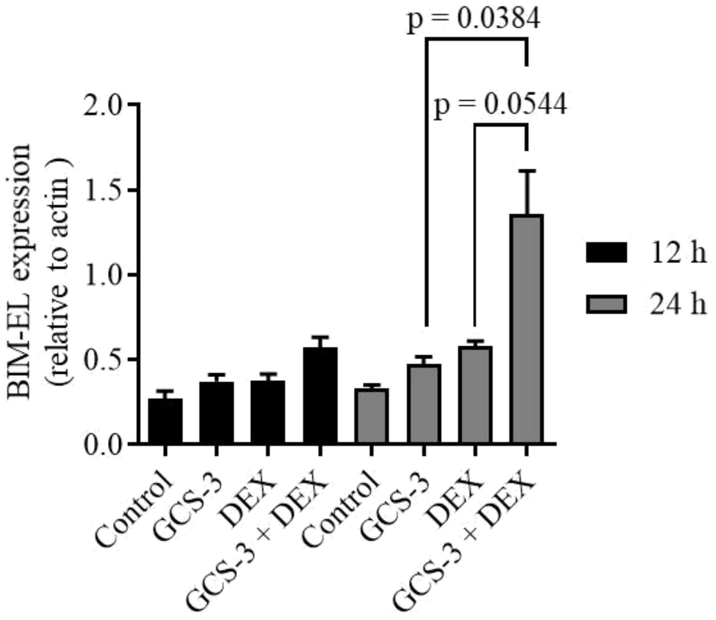

Supplementary Figure S13. Effect of GCS-3/dexamethasone on BIM_EL expression. ALL-19 xenograft cells were treated with 10 µM dexamethasone (DEX), 10 µM GCS-3, or both in combination for 12 h and 24 h. Equal amounts of protein (20 µg) were immunoblotted for BIM and actin (loading control). BIM-EL expression relative to loading control is shown. Each data point represents the mean ± SEM of four independent experiments. Significance was calculated using unpaired t-test with Welch's correction**.**

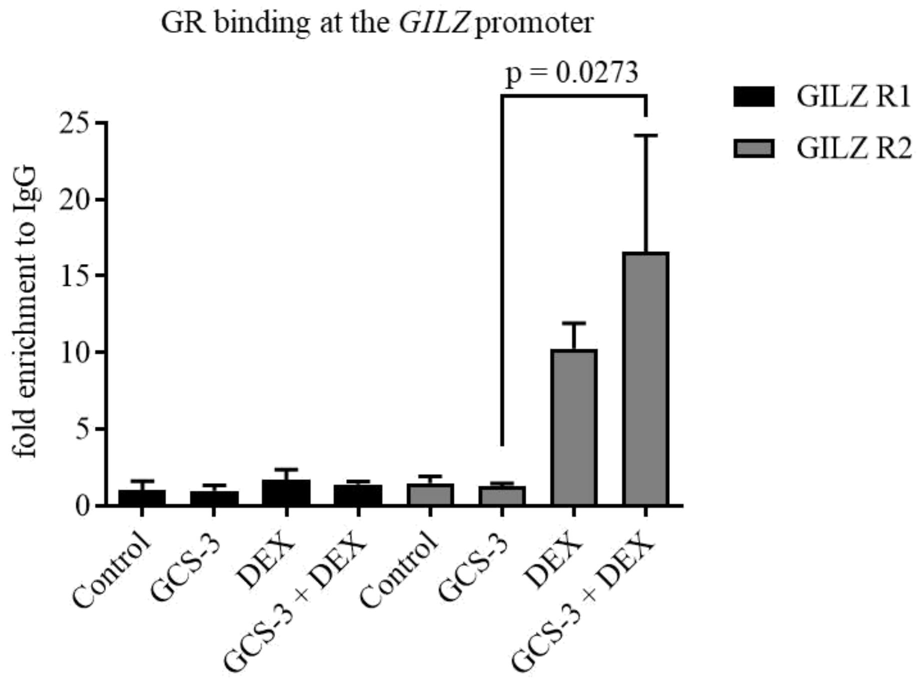

Supplementary Figure S14. GR binding at *GILZ*. Conventional ChIP of GR binding at GILZ R1/2. ALL-19 xenograft cells were treated with 10 µM dexamethasone, 10 µM GCS-3, or both in combination for 8 h. Fold change was calculated relative to the IgG control. Each data point represents the mean ± SEM of three independent experiments. Significance was calculated using unpaired t-test with Welch’s correction.

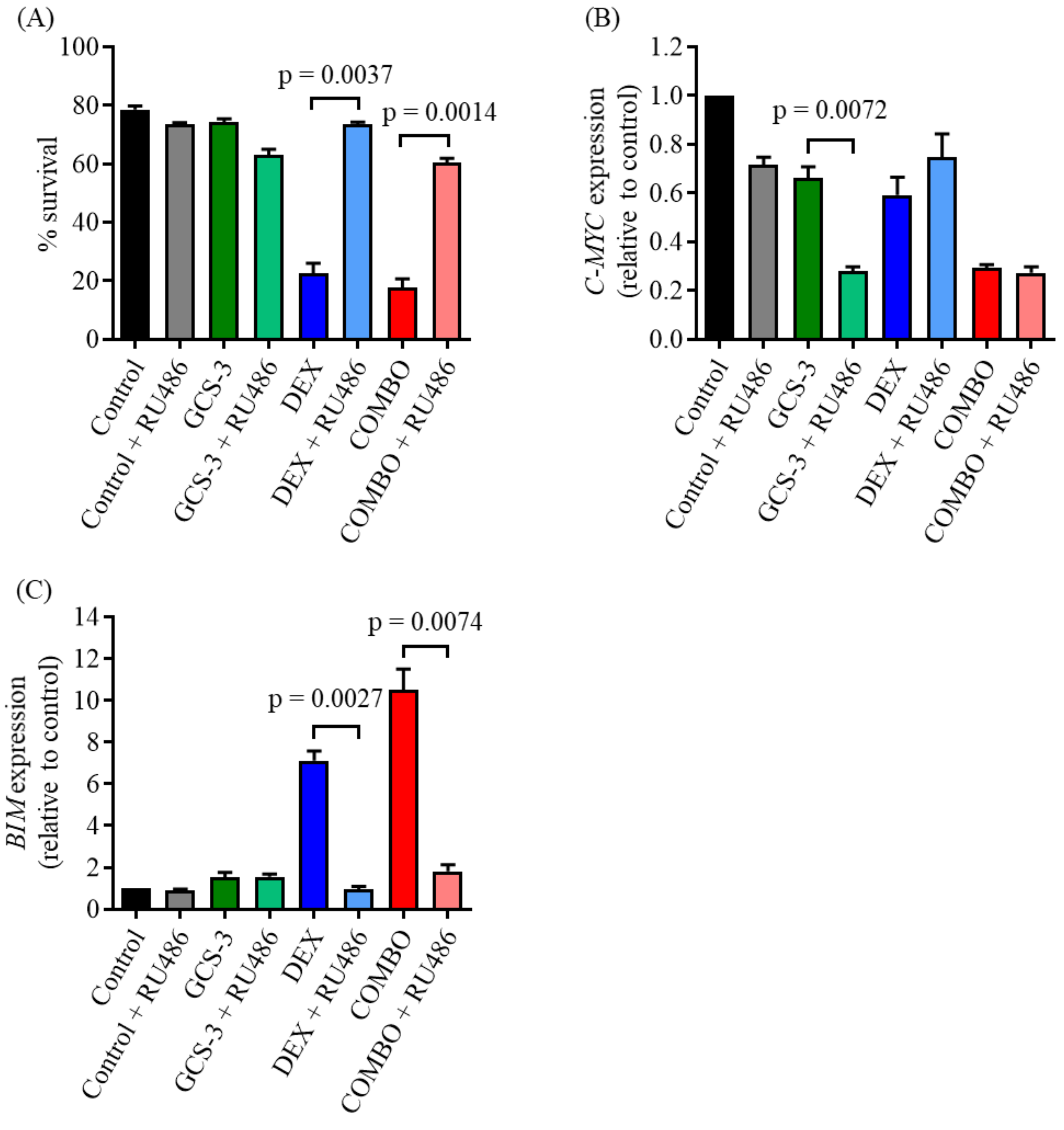

Supplementary Figure S15. *BIM* upregulation correlates with GCS-3/dexamethasone efficacy in ALL-54. (A-C) ALL-54 cells were pre-treated with 1 µM RU486, then treated with 10 µM GSC-3, 60 nM dexamethasone and the combination for 24 h (qRT-PCR) or 48 h (cell sensitivity). (C) Cell sensitivity was assessed by flow cytometry. (D) *C-MYC* and (E) *BIM* mRNA expression was analysed by qRT-PCR and calculated relative to the control. Each data point represents the mean ± SEM of three independent experiments. Significance was calculated using the unpaired t-test with Welch's correction.
